# Maternal and neonatal outcomes after infection with monkeypox virus clade I during pregnancy in DR Congo: a pooled, prospective cohort study

**DOI:** 10.1016/S0140-6736(25)02309-8

**Published:** 2026-01-17

**Authors:** Emmanuel Hasivirwe Vakaniaki, Isaac Barhishindi, Ange Mubiala, Espoir Bwenge Malembaka, Lydia Braunack-Mayer, Bruce Nganga, Sabin Sabiti Nundu, Isabel Brosius, Stefanie Bracke, Eugene Bangwen, Elise De Vos, Robert Colebunders, Mireille Ngale, Gabriel Kayembe, Christian Tshongo, Ali Dilu, Celestin Tshimanga, Jean-Luc Biampata, Patrick Musole Bugeme, Nsengi Ntamabyaliro, Bruce Kirenga, Misaki Wayengera, Joseph Nelson Siewe Fodjo, David Lupande Mwenebitu, Anne W Rimoin, Tony Wawina-Bokalanga, Koen Vercauteren, Daniel Mukadi-Bamuleka, Jean-Jacques Muyembe-Tamfum, Susanne Krasemann, Jason Kindrachuk, Andrew S Azman, Veronique Nussenblatt, Ian Crozier, Lori E Dodd, Olivier Tshiani-Mbaya, MBOTE-SK Consortium, Steve Ahuka-Mundeke, Adrienne Amuri-Aziza, Jean-Claude Makangara-Cigolo, Fiston Mpinda-Isekusu, Eddy Kinganda-Lusamaki, Raphaël Lumembe-Numbi, Yves Mujula, Papy Munganga, Jean-Claude Tshomba, Sarah Houben, Christophe Van Dijck, Nicole A. Hoff, Sydney Merritt, Martine Peeters, Richard Kojan, Léandre Mutimbwa-Mambo, Jenestin Babingwa Munga, Steeven Bilembo Kitwanda, Divin Mazambi Mambo, Franklin Mweshi Kumbana, James Wakilongo Zangilwa, Cris Kacita, PREGMPOX Consortium, Esto Bahizire, Wyvine Bapolisi, Bertin C. Bisimwa, Arsene Daniel Nyalundja, Mudarshiru Bbuye, Claudine Mukashyaka, Marina Saleeb, Yves Jacquemyn, Samir Kumar-Singh, Osama A.A. Mohamed, PALM007 Consortium, Rosine Ali, Michael Kombozi Basika, Esaie Kindombe, Mays Kisala, Augustin Ibanda, Baudouin Matiba Baudouin, Gael Mukendi, Patrick Mutombo, Tona Lutete, Mariano Lusakibanza, Yves Lula, Patricia Kabuni, Hippy Lonza, Sylvain Mulumba, Henri Mutomboh, Yves-Ninon Kumuamba, Jules Alonga, Claude Shosongo, Katherine Cone, Uvira Study Group, Trust Faraja Mukika, Patrick Kazuba Bugale, Salomon Mashupe Shangula, Levi Bugwaja, Stephanie Ngai, Jules Jackson, Nicola Low, Patrick D M C Katoto, Placide Mbala-Kingebeni, Laurens Liesenborghs

**Affiliations:** a Institut National de Recherche Biomédicale, Kinshasa, DR Congo; b Department of Clinical Sciences, Institute of Tropical Medicine, Antwerp, Belgium; c Department of Microbiology, Immunology and Transplantation, KU Leuven, Leuven, Belgium; d Center for Tropical Diseases and Global Health, Catholic University of Bukavu, Bukavu, DR Congo; e Faculty of Medicine, Catholic University of Bukavu, Bukavu, DR Congo; f Global Health Institute, University of Antwerp, Antwerp, Belgium; g Department of Epidemiology, Johns Hopkins Bloomberg, School of Public Health, Baltimore, MD, USA; h Institute of Social and Preventive Medicine, University of Bern, Bern, Switzerland; i The Kids Research Institute Australia, Nedlands, WA, Australia; j Department of Pharmacology, Faculty of Medicine, University of Kinshasa, Kinshasa, DR Congo; k Makerere Lung Institute, Makerere University College of Health Sciences, Mulago Hospital, Kampala, Uganda; l Department of Epidemiology, Jonathan and Karin Fielding School of Public Health, University of California, Los Angeles, CA, USA; m Service de Microbiologie, Département de Biologie Médicale, Cliniques Universitaires de Kinshasa, Université de Kinshasa, Kinshasa, DR Congo; n Rodolphe Mérieux Institut National de Recherche Biomédicale, Goma Laboratory, Goma, DR Congo; o Institute of Neuropathology, University Medical Center Hamburg-Eppendorf, Hamburg, Germany; p National Microbiology Laboratory Branch, Public Health Agency of Canada, Winnipeg, MB, Canada; q Geneva Centre for Emerging Viral Diseases and Division of Tropical and Humanitarian Medicine, Geneva University Hospitals, Geneva, Switzerland; r Institute of Global Health, University of Geneva, Geneva, Switzerland; s National Institute of Allergy and Infectious Diseases, Bethesda, MD, USA; t Clinical Monitoring Research Program Directorate, Frederick National Laboratory for Cancer Research, Frederick, MD, USA; u Division of Epidemiology and Biostatics, Cochrane South Africa, South African Medical Research Council, Cape Town, South Africa; v Department of Global Health, Faculty of Medicine, Stellenbosch University, Stellenbosch, South Africa; w South African National Bioinformatics Institute, University of the Western Cape, Cape Town, South Africa; x Department of Virology, Graduate School of Medicine, Osaka Metropolitan University, Osaka, Japan

## Abstract

**Background:**

Monkeypox virus (MPXV) has been linked to vertical transmission, but systematic data are scarce. We aimed to describe the sociodemographic, clinical, and virological characteristics and assess the frequency and determinants of adverse outcomes in pregnant women with MPXV clade I infection.

**Methods:**

In this prospective cohort study, we pooled data from three cohort studies (MBOTE-SK, PREGMPOX, and Uvira mpox) and one randomised controlled trial (PALM007) conducted in the South Kivu, Maniema, and Sankuru provinces of DR Congo between Dec 29, 2022, and June 20, 2025. Pregnant women and adolescent girls with a PCR-confirmed diagnosis of mpox were followed up throughout hospitalisation for mpox, delivery, and until discharge during the postpartum period. We extracted data on sociodemographic characteristics, MPXV exposure, clinical and obstetric presentation, and laboratory results. In a univariable analysis, we examined factors associated with the following adverse outcomes: spontaneous or missed abortion (<20 weeks of gestation), stillbirth (≥20 weeks of gestation), preterm birth (<37 weeks of gestation), live birth of a neonate with macroscopic mpox-like lesions, early (first 7 days) neonatal death, congenital anomaly, or maternal death (during pregnancy or discharge postpartum).

**Findings:**

We collected data from 89 pregnant women in the first (25 [28%]), second (31 [35%]), and third (33 [37%]) trimesters across all four studies: MBOTE-SK (36 [40%]), PREGMPOX (24 [27%]), PALM007 (25 [28%]), and Uvira mpox (four [4%]). All participants recovered from mpox; no maternal deaths were reported. During hospitalisation for mpox, fetal loss was reported in 17 (19%) women. Final pregnancy outcomes were known for 69 (78%) participants; adverse outcomes were reported in 35 (51%) women (95% CI 38–63), including fetal loss in 31 (45%; 95% CI 33–57; 16 [52%] spontaneous abortions, four [13%] missed abortions, and 11 [35%] stillbirths). Of the 38 live births, four neonates had congenital mpox-like lesions; one infant died a few hours after birth. No preterm births or congenital abnormalities were recorded. MPXV infection during the first trimester was associated with a higher risk of adverse pregnancy outcomes than during the second (risk ratio [RR] 0·6 [95% CI 0·4–0·9]) and third (0·2 [0·1–0·4]) trimesters (p=0·0008). Adverse outcomes were also associated with high viral load in skin lesions (PCR cycle threshold ≤30; RR 3·5 [95% CI 1·0–12·3]; p=0·045), direct sexual contact with the index case (1·6 [1·1–2·4]; p=0·026), positive HIV status (2·0 [1·4–2·9]; p=0·0002), and the presence of genital lesions (1·9 [1·1–3·2]; p=0·025).

**Interpretation:**

MPXV clade I infection in pregnancy is associated with a high risk of fetal loss and congenital infection, particularly during the first trimester. Targeted preventive and clinical strategies are urgently needed to protect pregnant women and their infants in settings that are endemic and epidemic for mpox.

**Funding:**

The European and Developing Countries Clinical Trials Partnership, the Belgian Directorate-General Development Cooperation and Humanitarian Aid, the Swiss National Science Foundation, the Research Foundation–Flanders, the Gates Foundation, the Intramural Research Program of the National Institutes of Health, and the National Cancer Institute.

## Introduction

Since 2023, a large epidemic of mpox has affected the African continent, particularly DR Congo. This epidemic has been triggered by a combination of the increasing zoonotic spillover of monkeypox virus (MPXV) clade Ia^1^ and the emergence of two strains associated with sustained human-to-human transmission (clade Ia/sh2024 and clade Ib/sh2023).^2^, ^3^ Given that sexual contact is one of the main transmission modes of MPXV, mpox often affects individuals of reproductive age, raising concerns around the maternal and neonatal effects of infection during pregnancy.^2^, ^4^

**Research in context**

**Evidence before this study**

Monkeypox virus (MPXV) infection in pregnancy has been linked to vertical transmission and adverse pregnancy outcomes. We searched PubMed, Google Scholar, medRxiv, and bioRxiv for articles published in English from database inception to Sept 12, 2025, using the following terms: (“monkeypox” OR “mpox” OR “monkeypox virus”) AND (“pregnancy” OR “pregnancy outcome” OR “perinatal” OR “pregnancy complications” OR “pregnant people” OR “vertical transmission” OR “placenta”). We also searched websites and publications from WHO and the US Centers for Disease Control and Prevention. Our search identified 52 publications, of which 18 reported primary clinical data from 16 studies, three reported preclinical data, and four were systematic reviews; the other 27 publications were narrative reviews, guidelines, letters, or editorial commentaries without primary data. Seven publications from 16 studies were from DR Congo, where MPXV clade I is endemic. These publications described 64 pregnancies, with adverse outcomes reported in 21 (70%) of 30 pregnancies with known outcomes. The earliest report of mpox in pregnancy was published in 1988 in Zaire (present day DR Congo). A woman who presented with mpox in the third trimester gave birth to a premature infant with a generalised mpox rash who died 6 weeks later. Another study, conducted between 2007 and 2011 in an area of DR Congo endemic for MPXV clade Ia, reported MPXV infection in four pregnancies, of which two resulted in spontaneous abortion and one in a stillbirth with visible fetal lesions. Histopathological evidence confirmed vertical transmission. Three studies have reported pregnancy outcomes in the South Kivu province, where MPXV clade Ib/sh2023 was first identified in 2023. In a study of 21 pregnant women with mpox, known pregnancy outcomes for six women included three fetal deaths and one full-term infant with mpox skin lesions who died shortly after birth. A report on three cases, one from each trimester, provided histological and molecular evidence on the vertical transmission of MPXV. An additional report from the same region documented 14 pregnancies with eight adverse outcomes. An mpox surveillance-based study conducted between October, 2023, and September, 2024, documented pregnancy loss in two of 21 reported pregnancies.

**Added value of this study**

In this pooled, prospective cohort study, we collected data on 89 pregnant women from three provinces across DR Congo, including two where MPXV clade Ia is in circulation and one where clade Ib is in circulation. This study design provides evidence on the adverse outcomes of infection with mpox clade I in pregnancy, which is less biased and more precise than the conclusions of case reports and case series. The geographical coverage of the study, covering areas affected by both clade Ia and Ib/sh2023 MPXV, makes these findings more generalisable than studies from a single location in DR Congo. By studying women with mpox diagnosed at all stages of pregnancy and following them up until delivery, this study shows a higher risk of adverse outcomes with mpox infection during the first trimester than during the second or third trimester. The study identified factors that clinicians and researchers can use for future monitoring and investigation of pregnant women with mpox clade I infection.

**Implications of all the available evidence**

MPXV clade I infection is associated with a substantial risk of adverse pregnancy outcomes, particularly when infection occurs during the first trimester. MPXV acquired during pregnancy should be regarded as a congenital pathogen, with outcomes resembling those of other congenital viral infections. Pregnant women and women of childbearing age should be prioritised for preventive and therapeutic interventions, including vaccination. Long-term studies are needed to assess the developmental consequences of congenital mpox.

Vertical transmission of MPXV was first reported in 1988, when a mother with mpox delivered a premature infant with a generalised rash who died aged 6 weeks.^5^ In the subsequent decades, evidence on the effects of mpox in pregnancy has increased but remains fragmented. In a cohort study conducted from 2007 to 2011 in the Sankuru province of DR Congo, pregnancy loss was documented in three of four pregnant women infected with MPXV clade I.^6^, ^7^ Additionally, MPXV antigens were detected in fetal placental tissue.^7^ In a cohort of patients with MPXV clade Ib/sh2023 infection in the South Kivu province in 2024, four adverse pregnancy outcomes were documented among six women.^4^ Clinical, molecular, and histopathological evidence from three women showed the vertical transmission of MPXV clade Ib/sh2023 across all trimesters.^8^ In a retrospective analysis of surveillance data on mpox from DR Congo, two of 21 recorded pregnancies ended in fetal loss.^9^ Adverse pregnancy outcomes have also been documented during outbreaks of MPXV clade IIb in Nigeria between 2017 and 2018; and in the USA, Colombia, Nigeria, and Spain during the 2022–23 global mpox outbreak.^10^, ^11^, ^12^, ^13^, ^14^, ^15^

Although these case series described various outcomes following MPXV infection in pregnancy, large-scale studies are needed to estimate the proportions of specific pregnancy outcomes and to identify factors associated with these outcomes. We aimed to describe the sociodemographic, clinical, and virological characteristics and assess the frequency and determinants of adverse outcomes in pregnant women with MPXV clade I infection.

## Methods

### Study design and participants

In this prospective cohort study, we pooled data from four studies conducted in three provinces across DR Congo between Dec 29, 2022, and June 20, 2025. We included women and adolescent girls who were pregnant at the time of PCR-confirmed diagnosis of mpox. The four studies were three prospective cohort studies (MBOTE-SK, PREGMPOX, and Uvira mpox), which recruited participants from areas with MPXV clade Ib/sh2023 in circulation, and one clinical trial (PALM007),^16^ which enrolled participants from areas endemic for MPXV clade Ia.^17^, ^18^

The MBOTE-SK study (NCT0665264) is an ongoing clinical cohort study to characterise MPXV clade Ib infection in the South Kivu province of DR Congo.^4^ The study has been enrolling patients with mpox at Kamituga General Hospital since May, 2024. Skin swabs are tested on site for the presence of orthopoxvirus DNA by use of GeneXpert (Cepheid, Sunnyvale, CA, USA). A positive MPXV result is defined as a PCR cycle threshold (Ct) value of less than 40.^4^ Sociodemographic characteristics, exposure to MPXV clade Ib, medical history, and clinical features are collected in an electronic case report form, with a dedicated form detailing participants' obstetric history and pregnancy outcomes. Pregnant women are followed up daily during hospitalisation, on days 28 and 59 after enrolment, and at the time of delivery or in the case of suspected complications.

The PREGMPOX study (NCT06442501) is an ongoing cohort study of pregnant patients with mpox in four health zones across the South Kivu province (Nyangezi, Kadutu, Miti-Murhesa, and Nyantende) that started in November, 2024. Skin lesions and oropharyngeal swabs are tested at the Université Catholique de Bukavu (UCB) laboratory with conventional PCR. A PCR sample is classified as positive for MPXV when an amplification band is detected during electrophoresis. The study uses the same electronic case report form as the MBOTE-SK study. Participants with moderate or severe mpox are admitted to hospital to undergo daily clinical assessments until discharge. Women with mild disease are followed up weekly as outpatients until recovery. Follow-up visits are scheduled on days 21 and 56 after admission, between 36 and 37 weeks of gestation, at delivery, and in the case of any complications.

Starting in May 2024, the Uvira mpox study is an ongoing cohort study aimed at establishing the clinical, epidemiological, and immunological characteristics of mpox. Individuals with suspected mpox are recruited from Uvira General Hospital in the South Kivu province. Skin lesions or oropharyngeal swabs are tested at the Rodolphe Mérieux INRB Laboratory in Goma with the RADI FAST Mpox Detection Kit (KH Medical; Pyeongtaek, South Korea). Ct values of less than 40 are considered to be positive for MPXV. Data on demographics, exposures, clinical presentation, and outcomes are collected in an electronic case report form. Pregnant women are followed up throughout hospitalisation and after discharge via home visits and at delivery by a study physician or nurse.

The PALM007 trial (NCT05559099) was a phase 2, double-blinded, randomised controlled trial evaluating tecovirimat for treating mpox between October, 2022, and July, 2024, at Tunda General Hospital in the Maniema province and Kole General Hospital in the Sankuru province. The main results have been published.^16^ Participants of all ages, including pregnant women, with PCR-confirmed mpox were randomly assigned (1:1) to either placebo or tecovirimat (three oral capsules of 200 mg twice a day for 14 days for participants weighing between 40 kg and 120 kg).^16^ PCR testing was done on site with the RADI FAST Mpox Detection Kit, and a Ct value of less than 40 was considered positive for MPXV. All participants were hospitalised for at least 14 days and had follow-up visits on days 28 and 59 after enrolment. Pregnant individuals were followed up throughout their pregnancy as part of pharmacovigilance.

The MBOTE-SK study was approved by the ethics committee at University of Kinshasa (ESP/CE/78B/2024, ESP/CE/19/2025) and the institutional review board at the Institute of Tropical Medicine (1754/24). The PREGMPOX study was approved by the ethics committee at the University of Antwerp (B3002024000174) and UCB (UCB/CIES/NC/022/2024). The Uvira mpox study was approved by the institutional review board at Johns Hopkins Bloomberg School of Public Health (IRB00030442) and the ethics committee at UCB (UCB/CIES/NC/019/2024). The PALM007 trial was approved by the ethics committee at University of Kinshasa (ESP/CE/112/2022) and the Congolese Pharmaceutical Regulatory Authority (MSPHP.1253/P/DKK/014/2022). Study clinicians explained the study procedures in local languages using a context-adapted explanation. The MBOTE-SK study and PALM007 trial used health promoters to counsel participants, whereas the PREGMPOX and Uvira mpox studies collaborated with the risk communication and community engagement pillar of local health authorities. All participants provided written informed consent.

### Procedures

Key investigators of the four studies (LL, PM-K, SSN, RC, JNSF, PDMCK, EBM, ASA, VN, and OT-M) organised the collection of data. The investigators assigned one researcher for each study (EHV, IBa, BN, and EBM) to extract data from the database of the individual studies. We created a database (Microsoft Excel) to harmonise information from variables in the four studies and to record the de-identified data for all participants with available data. The database recorded sociodemographic characteristics (ie, age and occupation), MPXV exposure (ie, type of exposure and contact with index case), medical and obstetric history (including vaccination history), clinical and obstetric presentation, virological test results (eg, blood, skin and oropharyngeal swabs), co-infections (ie, HIV, syphilis, or malaria), and pregnancy outcomes (ie, delivery of a healthy neonate or an adverse outcome). Gestational age was based on either antenatal care records or last menstrual period. The WHO severity score, based on lesion count, was used to classify severity of mpox in participants as mild (<25 lesions), moderate (25–99 lesions), severe (100–250 lesions), or grave (>250 lesions).^19^ Adverse outcomes were spontaneous abortion (<20 weeks of gestation), missed abortion (<20 weeks of gestation), stillbirth (≥20 weeks of gestation^20^), preterm birth (<37 weeks of gestation), maternal death (during pregnancy or postpartum follow-up until hospital discharge), live birth of a neonate with macroscopic mpox-like skin lesions, early neonatal death (within the first 7 days), and congenital anomalies. In all studies, the presence or absence of congenital anomalies was based on clinical examination of the neonate.

All participants were identified as women by the investigators. Given that all participants were from DR Congo, data on ethnicity were not considered relevant and, therefore, not recorded.

### Statistical analysis

All statistical analyses followed a prespecified analysis plan (appendix pp 14–32). The sample size was based on the amount of available data. The primary aim was to describe the clinical characteristics of MPXV infection during pregnancy and to estimate the frequency of adverse outcomes. Continuous variables are presented as median (IQR) values; categorical variables as counts and percentages.

As an exploratory objective, we assessed factors that were potentially associated with adverse pregnancy outcomes based on previous case series and field observations.^6^, ^7^, ^8^, ^9^, ^10^, ^11^, ^12^, ^13^, ^14^, ^15^ Selected factors were age, trimester at diagnosis of mpox, severity (according to the WHO severity score^19^), Ct values of skin lesions or oropharyngeal swabs, type of contact with a suspected or confirmed person with mpox, HIV infection status, co-infection with malaria, presence of fever, presence of genital skin lesions, and suspected MPXV clade (attributed based on health zone).^18^ We used a univariable Poisson regression model with robust standard errors to calculate risk ratios (RRs) and 95% CIs for each factor. All statistical analyses were performed with R (version 4.4.2).

### Role of the funding source

The funders of the study had no role in study design, data collection, data analysis, data interpretation, or writing of the report.

## Results

We collected data from 89 pregnant women with PCR-confirmed mpox across all four included studies: MBOTE-SK (36 [40%] participants), PREGMPOX (24 [27%]), PALM007 (25 [28%]), and Uvira mpox (four [4%]; table 1; appendix p 7). Median participant age was 24 years (IQR 20–30; range 15–42). Most participants were enrolled in South Kivu (64 [72%]), followed by Maniema (17 [19%]) and Sankuru (eight [9%]). 64 (72%) participants were diagnosed in areas affected by MPXV clade Ib/sh2023 and 25 (28%) in regions affected by MPXV clade Ia. Reported primary professional activity included housework (35 [39%]), farming (24 [27%]), commercial activities (18 [20%]), and sex work (four [4%]).

**Table 1:** Study settings and participant demographics, MPVX exposure characteristics, and medical history

		**Participants (n=89)**
**Study settings**		
Original study		
	MBOTE-SK	36 (40%)
	PREGMPOX	24 (27%)
	PALM007	25 (28%)
	Uvira mpox	4 (4%)
Province		
	South Kivu	64 (72%)
	Maniema	17 (19%)
	Sankuru	8 (9%)
Suspected MPXV clade*		
	Clade Ib/sh2023	64 (72%)
	Endemic zoonotic clade Ia	25 (28%)
**Participant demographics**		
Age, years		24 (20–30)
Age group, years		
	≤18	14 (16%)
	19–34	61 (69%)
	≥35	14 (16%)
Primary occupation		
	Home maker	35 (39%)
	Farmer	24 (27%)
	Businesswoman	18 (20%)
	Sex worker	4 (4%)
	Unemployed	4 (4%)
	Student	1 (1%)
	Other	3 (3%)
**MPXV exposure characteristics**		
Exposure in past 3 weeks		
	Hunting	1/88 (1%)
	Consumption of rodents	4/87 (5%)
	Manipulation of wildlife meat	10/86 (12%)
	Consumption of wildlife meat	4/85 (5%)
	Contact with another mpox case	57/73 (78%)
Type of contact with index case†		
	Direct, skin-to-skin, non-sexual contact	35/55 (64%)
	Sexual contact	20/55 (36%)
	Fomite	0
**Medical history**		
Childhood smallpox vaccination		0
Mpox vaccination with MVA-BN		3/85 (4%)
Coinfection‡		
	HIV	3/35 (9%)
	Syphilis	0
	Malaria	12/86 (14%)
Treated with tecovirimat§		8 (9%)

Data are n (%), median (IQR), or n/N (%). Denominators represent the number of participants with available data. MPXV=monkeypox virus.

* Attributed based on health zone. According to genomic sequencing analyses, no co-circulation of clade Ib/sh2023 and zoonotic clade Ia has been reported in the study areas as of May, 2025.

† Classified as the main exposure only.

‡ Diagnosed as part of routine clinical care, either during hospitalisation or prenatal care.

§ Participants in the PALM007 trial, including pregnant women, were randomly assigned (1:1) to receive either placebo or tecovirimat (three 200 mg capsules twice daily for 14 days for those weighing 40–120 kg).

Reported exposure to known or potential animal reservoirs in the 3 weeks before disease onset was uncommon among participants with available data: ten (12%) of 86 women reported manipulation of dead wild animals, four (5%) of 85 indicated consuming wildlife, four (5%) of 87 reported eating rodents, and one (1%) of 88 participants reported hunting activities. By contrast, in the 3 weeks preceding disease onset, 57 (78%) of 73 participants reported contact with a suspected or confirmed individual with mpox, primarily through direct, non-sexual, skin-to-skin contact (35 [64%] of 55) or sexual contact (20 [36%]).

At diagnosis of mpox, no participant reported childhood smallpox vaccination; three (4%) of 85 participants with available data reported vaccination against mpox with MVA-BN (table 1). Among 86 participants tested for malaria, 12 (14%) were positive. HIV and syphilis testing were performed infrequently; three (9%) of 35 individuals tested for HIV had positive status, whereas none of the 32 participants tested for syphilis had a positive result. Eight (9%) of all 89 participants had been treated with tecovirimat during acute illness as part of the PALM007 trial.

Median time from symptom onset to study enrolment was 7 days (IQR 4–10). The most frequently reported systemic symptoms were fatigue, myalgia or arthralgia, headache, sore throat or dysphagia, anorexia, and fever (table 2). The most common symptoms associated with mpox lesions were itching and pain (table 2). Genitourinary symptoms included dysuria and genital pain.

**Table 2:** Clinical presentation and outcomes of pregnant women with mpox

			**Participants (n=89)**
Time between symptom onset and enrolment, days			7·0 (4·0–10·0)
Symptoms			
	Fever		33/88 (38%)
	Fatigue		55/88 (63%)
	Itching		77/89 (87%)
	Myalgia or arthralgia		48/88 (55%)
	Pain in lesions		71/89 (80%)
	Headache		43/89 (48%)
	Sore throat or dysphagia		41/89 (46%)
	Cough		19/89 (21%)
	Anorexia		39/85 (46%)
	Abdominal pain		24/85 (28%)
	Vomiting or nausea		24/89 (27%)
	Diarrhoea		10/89 (11%)
	Dysuria		28/85 (33%)
	Genital pain		30/89 (34%)
	Rectal pain		8/89 (9%)
	Eye pain		9/85 (11%)
	Convulsion or altered consciousness		1/83 (1%)
Signs			
	Presence of skin lesions		89/89 (100%)
	Number of lesions		62·0 (18·5–179·2)
	WHO severity score by lesion count		
		Mild (<25)	27/87 (31%)
		Moderate (25–99)	26/87 (30%)
		Severe (100–250)	20/87 (23%)
		Grave (>250)	14/87 (16%)
	Type of skin lesion		
		Macule	13/85 (15%)
		Papule	31/87 (36%)
		Vesicle	69/87 (79%)
		Small pustule	52/84 (62%)
		Umbilicated pustule	31/83 (37%)
		Ulcerated lesion	16/83 (19%)
		Crust	16/83 (19%)
		Scar	14/84 (17%)
	Genital skin lesions		46/84 (55%)
	Genital oedema		23/81 (28%)
	Rectal lesions		11/83 (13%)
	Lymphadenopathy		53/83 (64%)
Virological test results			
	MPXV PCR-positive skin lesion		89/89 (100%)
		Ct value of skin lesion swab	20·5 (18·1–25·0)
	MPXV PCR-positive oropharyngeal swab		30/34 (88%)
		Ct value of oropharyngeal swab	28·3 (23·8–31·2)
	MPXV PCR-positive blood sample*		12/26 (46%)
Clinical outcomes during hospitalisation			
	Duration of hospital stay, days		13·0 (7·0–15·0)
	Maternal death		0

Data are median (IQR) or n/N (%). Denominators represent the number of participants with available data. Ct=cycle threshold. MPXV=monkeypox virus.

* Only one Ct value available: 33·1.

All participants had active skin lesions at the time of diagnosis. Of the 87 participants that could be classified according to WHO severity score, 27 (31%) had mild disease, 26 (30%) had moderate disease, 20 (23%) had severe disease, and 14 (16%) had grave disease. Genital skin lesions were documented in 46 (55%) of 84 and genital oedema in 23 (28%) of 81 participants with available data. Lymphadenopathy was reported in 53 (64%) of 83 participants. Median duration of hospital stay for mpox was 13 days (IQR 7–15; table 2), primarily determined by local isolation policies. No participant died during hospitalisation.

All participants tested positive for MPXV DNA by skin swab PCR (median Ct value 20·5 [IQR 18·1–25·0]; table 2). Oropharyngeal swabs were PCR-positive in 30 (88%) of 34 and blood samples were PCR-positive in 12 (46%) of 26 participants sampled.

All participants had singleton pregnancies and were enrolled across all gestational trimesters (table 3). Only eight (13%) of 60 participants with available data reported attending prenatal consultation. Among women with available data, 16 (19%) of 83 reported a history of miscarriage, four (6%) of 67 reported having a previous stillbirth, and one (2%) of 67 reported a previous preterm delivery. Documented pregnancy complications during the current pregnancy of 57 participants with available data included preterm pre-labour rupture of membranes (four [7%]) and polyhydramnios (three [5%]). No participant was diagnosed with hypertension or gestational diabetes.

**Table 3:** Obstetric history and outcomes in pregnant women with mpox

		**Participants (n=89)**
**Gestational trimester at the time of mpox diagnosis (weeks of gestation)**		
First (1–13)		25/89 (28%)
Second (14–27)		31/89 (35%)
Third (≥28)		33/89 (37%)
**Obstetric history**		
Female genital mutilation		0
Number of previous pregnancies		
	0	18/84 (21%)
	1	8/84 (10%)
	2	12/84 (14%)
	≥3	46/84 (55%)
Previous caesarean section		15/83 (18%)
Previous miscarriage		16/83 (19%)
Previous premature delivery		1/67 (2%)
Previous stillbirth		4/67 (6%)
Previous birth of neonate with congenital anomalies		0
Attended prenatal consultation		8/60 (13%)
Diagnosed complications during current pregnancy*		
Gestational diabetes		0
Hypertension		0
Polyhydramnios		3/57 (5%)
Preterm pre-labour rupture of membranes (<37 weeks of gestation)		4/57 (7%)
Pregnancy complications during hospital admission for mpox		18/89 (20%)
	Spontaneous abortion	11/89 (12%)
	Missed abortion	3/89 (3%)
	Stillbirth	3/89 (3%)
	Vaginal bleeding and abdominal pain without pregnancy loss	1/89 (1%)
**Pregnancy outcomes**		
Participants with available outcome data		69/89 (78%)
Delivery of healthy neonate		34/69 (49%)
Adverse outcome		35/69 (51%)
	Spontaneous abortion (<20 weeks of gestation)	16/69 (23%)
	Missed abortion (<20 weeks of gestation)	4/89 (5%)
	Stillbirth (>20 weeks of gestation)	11/69 (16%)
	Preterm live neonate (<37 weeks of gestation)	0
	Live neonate with macroscopic lesions	3/69 (4%)
	Live neonate with macroscopic lesions and neonatal death	1/69 (1%)
	Maternal death	0
	Neonate with congenital anomalies	0
MPXV PCR-positive placental swab		9/12 (75%)
	Ct value of placental swab	23·6 (20·9–32·2)
Duration from hospital admission to adverse outcome, days		
	Spontaneous abortion	11·0 (6·8–14·0)
	Missed abortion	20·5 (18·8–22·5)
	Stillbirth	14·0 (12·5–15·0)

Data are n/N (%) or median (IQR). Denominators represent the number of participants with available data. Ct=Cycle threshold. MPXV=monkeypox virus.

* Pregnancy complications occurring before discharge from the mpox treatment centre.

During acute hospitalisation for mpox, pregnancy complications occurred in 18 (20%) of all 89 participants, 17 (94%) of which resulted in fetal loss (11 [65%] spontaneous abortions, three [18%] missed abortions, and three [18%] stillbirths; table 3; figure). One participant had vaginal bleeding and abdominal pain without pregnancy loss. Final pregnancy outcomes were recorded for 69 (78%) of all 89 participants; the remaining 20 participants were lost to follow-up. Baseline characteristics between women with or without known pregnancy outcomes are outlined in the appendix (pp 8–9). Of the 69 participants with known outcomes, 34 (49%) had an uncomplicated delivery of a healthy infant. Adverse outcomes were reported in 35 (51%) women (95% CI 38–63), including fetal loss in 31 (45%; 95% CI 33–57; 16 [52%] spontaneous abortions, four [13%] missed abortions, and 11 [35%] stillbirths). Four (6%) of 69 women (95% CI 2–14) delivered a live neonate with macroscopic mpox-like lesions, of whom three survived and one died a few hours after birth from asphyxia. The diagnosis of mpox was confirmed in three of these four neonates through PCR; the neonate who died was not sampled. No maternal deaths, preterm births, or major clinical congenital abnormalities were recorded. The median duration from enrolment to adverse pregnancy outcome was 11·0 days (IQR 6·8–14·0) for spontaneous abortion, 20·5 days (18·8–22·5) for missed abortion, and 14·0 days (12·5–15·0) for stillbirth. MPXV DNA was detected in nine (75%) of 12 placental swabs tested.

**Figure:**
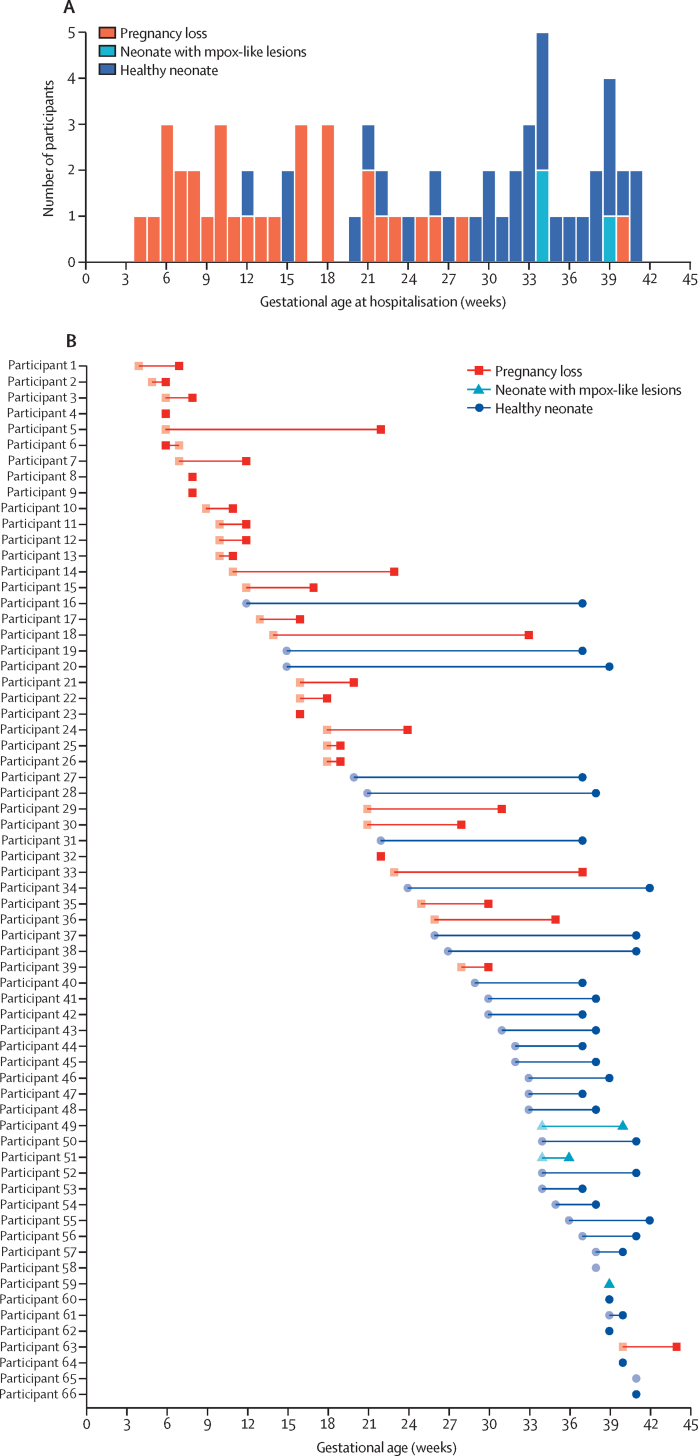
Pregnancy outcomes among 66 women with mpox by gestational age (A) Frequency of pregnancy outcomes (pregnancy loss, delivery of a neonate with mpox-like lesions, or delivery of a healthy neonate) stratified by gestational age at hospitalisation. (B) Individual participant timelines between hospitalisation for mpox (pale symbol) and delivery according to pregnancy outcome (dark symbol). Gestational age at delivery was unknown for two women. Three women with an unknown exact gestational age at hospitalisation were excluded.

In pregnancies with documented outcomes, adverse outcomes were more frequent when MPXV infection occurred in the first trimester (17 [94%] of 18 women) than in the second (13 [59%] of 22) or third trimester (five [17%] of 29; figure; appendix pp 10–11). In the univariable analysis, infection diagnosed during the first trimester was associated with an increased risk of adverse outcomes (table 4). Compared with the first trimester, this risk was lower in the second trimester (RR 0·6 [95% CI 0·4–0·9]) and third trimester (0·2 [0·1–0·4]; p=0·0008). In this analysis, adverse pregnancy outcomes were associated with direct sexual contact with the suspected index case (RR 1·6 [1·1–2·4]; p=0·026), the presence of genital lesions (1·9 [1·1–3·2]; p=0·025), high viral load in skin lesions (Ct value ≤30; 3·5 [1·0–12·3]; p=0·045), and positive HIV status (2·0 [1·4–2·9]; p=0·0002; table 4). We found no strong statistical evidence of an association between adverse pregnancy outcomes and co-infection with malaria during hospitalisation, maternal age, disease severity, viral load on oropharyngeal swab, or the presence of fever (table 4). We additionally found that outcomes were similar according to presumed MPXV clade, based on the area of study recruitment (table 4; appendix p 12).

**Table 4:** Factors associated with adverse pregnancy outcomes

		**Overall (n=69)**	**Healthy neonates (n=34)**	**Adverse outcomes (n=35)**	Risk ratio*(95% CI)	**p value**†
**Demographics**						
Maternal age group, years						
	≤18	10/69 (14%)	5/10 (50%)	5/10 (50%)	1 (ref)	0·48
	19–34	46/69 (67%)	20/46 (44%)	26/46 (57%)	1·1 (0·6–2·2)	..
	≥35	13/69 (19%)	9/13 (69%)	4/13 (31%)	0·6 (0·2–1·7)	..
Suspected MPVX clade						
	Clade Ia	24/69 (35%)	11/24 (46%)	13/24 (54%)	1 (ref)	0·67
	Clade Ib/sh2023	45/69 (65%)	23/45 (51%)	22/45 (49%)	0·9 (0·6–1·4)	..
**Gestational characteristics at diagnosis**						
Gestational age, weeks		23·5 (13·2–34·0)	33·0 (26·8–37·2)	15·0 (8·2–21·8)	..	..
	Data missing	3/69 (4%)	2/34 (6%)	1/35 (3%)	..	..
Trimester						
	First	18/69 (26%)	1/18 (6%)	17/18 (94%)	1 (ref)	0·0008
	Second	22/69 (32%)	9/22 (41%)	13/22 (59%)	0·6 (0·4–0·9)	..
	Third	29/69 (42%)	24/29 (83%)	5/29 (17%)	0·2 (0·1–0·4)	..
Type of MPXV exposure‡						
	Direct, non-sexual, skin-to-skin contact with index case	30/43 (70%)	14/30 (47%)	16/30 (53%)	1 (ref)	0·026
	Direct sexual contact with index case	13/43 (30%)	2/13 (15%)	11/13 (85%)	1·6 (1·1–2·4)	..
**Clinical presentation**						
Total lesion count		60·0 (13·0–164·5)	66·0 (9·0–183·0)	59·0 (25·0–150·0)	..	..
	Data missing	7/69 (10%)	5/34 (15%)	2/35 (6%)	..	..
WHO severity score by lesion count						
	Mild (<25)	22/67 (33%)	13/22 (59%)	9/22 (41%)	1 (ref)	0·69
	Moderate (25–99)	19/67 (28%)	6/19 (32%)	13/19 (68%)	1·7 (0·9–3·0)	..
	Severe (100–250)	16/67 (24%)	8/16 (50%)	8/16 (50%)	1·2 (0·6–2·5)	..
	Grave (>250)	10/67 (15%)	5/10 (50%)	5/10 (50%)	1·2 (0·6–2·7)	..
Fever						
	No	44/68 (65%)	21/44 (48%)	23/44 (52%)	1 (ref)	0·62
	Yes	24/68 (35%)	13/24 (54%)	11/24 (46%)	0·9 (0·5–1·5)	..
Genital skin lesions						
	No	28/64 (44%)	18/28 (64%)	10/28 (36%)	1 (ref)	0·025
	Yes	36/64 (56%)	12/36 (33%)	24/36 (67%)	1·9 (1·1–3·2)	..
Living with HIV						
	No	28/31 (90%)	14/28 (50%)	14/28 (50%)	1 (ref)	0·0002
	Yes	3/31 (10%)	0	3/3 (100%)	2·0 (1·4–2·9)	..
Co-infection with malaria						
	No	59/68 (87%)	30/59 (51%)	29/59 (49%)	1 (ref)	0·26
	Yes	9/68 (13%)	3/9 (33%)	6/9 (67%)	1·4 (0·8–2·3)	..
**Virological test results**						
Ct value of skin lesion swab		20·6 (18·0–25·1)	23·5 (19·0–35·6)	20·1 (17·5–21·7)	..	..
	Data missing	19/69 (28%)	14/34 (41%)	5/35 (14%)	..	..
Viral load of skin lesion swab						
	Low (Ct value >30)	10/50 (20%)	8/10 (80%)	2/10 (20%)	1 (ref)	0·045
	High (Ct value ≤30)	40/50 (80%)	12/40 (30%)	28/40 (70%)	3·5 (1·0–12·3)	..
Ct value of oropharyngeal swab		28·8 (23·7–31·5)	30·0 (27·7–33·4)	27·3 (23·1–31·0)	..	..
	Data missing	44/69 (64%)	24/34 (71%)	20/35 (57%)	..	..
Viral load of oropharyngeal swab						
	Low (Ct value >30)	11/25 (44%)	5/11 (45%)	6/11 (55%)	1 (ref)	0·63
	High (Ct value ≤30)	14/25 (56%)	5/14 (36%)	9/14 (64%)	1·2 (0·6–2·3)	..

Data are n/N (%) or median (IQR), unless otherwise indicated. Denominators represent the number of participants with available data. Ct=cycle threshold. MPXV=monkeypox virus.

* Calculated with Poisson regression with robust SEs.

† Fisher's exact test for categorical variables and Wilcoxon rank-sum test for continuous variables.

‡ Contact type classified as the main exposure only.

## Discussion

In this pooled cohort of pregnant women with MPXV clade I infection, adverse pregnancy outcomes occurred in around half of participants. Among 69 pregnancies with documented outcomes, 31 resulted in fetal loss. Of the 38 live births, four neonates showed signs of congenital mpox with macroscopic, mpox-like skin lesions, one of whom died. The risk of an adverse outcome was highest when infection occurred during the first trimester.

Because this study only involved women with PCR-confirmed mpox, the proportion of adverse outcomes attributable to vertical transmission of MPXV cannot be derived from these data. Accurate estimates of the background risk of pregnancy loss in the region are scarce. A multi-country cohort study involving over 270 000 pregnancies, including 6407 in DR Congo, estimated that around 3·3% of pregnancies in sub-Saharan Africa result in fetal loss (abortion or stillbirth).^21^ However, these data likely underestimate the risk of miscarriage. In European and North American cohorts, the pooled risk of miscarriage was 15·3%.^22^ Additionally, the 2021 GBD study estimated that 3·7% of pregnancies in DR Congo ended in stillbirth.^23^ All these estimates are substantially lower than the observed 45% frequency of fetal loss in this study. Together with the presence of mpox-like skin lesions in neonates, the detection of MPXV DNA in placental swabs, and previous histopathological evidence of vertical transmission, these observations indicate that a substantial proportion of the observed pregnancy complications were related to MPXV.

Our findings substantiate previous reports of vertical transmission of MPXV clade I, showing that complications are far from rare. However, data on infection with MPXV clade IIb in pregnancy remain scarce. Despite more than 100 000 cases during the global 2022–23 outbreak of mpox, women were rarely affected and few clade IIb cases in pregnancy have been documented. In a US national report of 23 pregnant women with mpox, four had pregnancy loss and two delivered neonates who developed the disease within 2 weeks of birth.^24^ In a cohort of ten pregnant Brazilian women with MPXV clade IIb infection, no vertical transmission was documented.^25^ However, these numbers are too small to draw conclusions on potential differences in vertical transmission between MPXV subclades. Interestingly, the proportion of adverse pregnancy outcomes in our pooled cohort is similar to that previously reported for variola virus. A retrospective analysis of outbreaks in the 19th and early 20th century involving 1074 pregnant women with smallpox showed a 39·9% rate of miscarriage and premature birth.^26^ Therefore, vertical transmission and adverse pregnancy outcomes seem to be a key characteristic of orthopoxvirus infections.

For other pathogens associated with vertical transmission, such as *Toxoplasma* and cytomegalovirus, the risk of transmission increases with gestational age, whereas risk of severe disease is highest early in pregnancy.^27^ In our pooled cohort, we also observed that fetal loss was associated with infection during the first trimester. Whether MPXV vertical transmission occurs more frequently at particular gestational ages is unclear. 24 (83%) women with infections during the third trimester delivered live neonates who seemed healthy, and no major congenital malformations were observed. However, we did not investigate whether vertical transmission had occurred in these healthy infants, and detailed paediatric investigations and longitudinal follow-up were not possible. Additional studies are needed to establish the frequency of vertical transmission and its potential association with long-term developmental problems in all neonates, especially those with lesions.

None of the pregnant women included in our pooled cohort died. This finding is compatible with the parent MBOTE-SK and PALM007 studies, which reported a maternal mortality rate of 0·5% and 1·7%, respectively.^4^, ^16^ Although our pooled cohort was too small to accurately estimate the case fatality ratio of women diagnosed with mpox in pregnancy who subsequently died of mpox, MPXV infection does not seem to greatly increase the risk of maternal mortality, despite the relative immunosuppression associated with pregnancy. With regard to standard, albeit few, measures of disease severity, 39% of participants in our cohort presented with more than 100 lesions, which is a similar frequency to that previously reported for non-pregnant adults with MPXV clade Ib infection from the same age group.^4^

To our knowledge, this study has yielded the largest dataset to date on MPXV clade I infection in pregnancy, but has important limitations. First, pooling data from different studies means that there were differences in the data available and high amounts of missing information for some variables, potentially biasing the results. Additionally, differences in the PCR platforms limit the comparability of viral load measurements. Second, the challenging field conditions and resource limitations in which data collection took place led to a considerable loss to follow-up after initial hospitalisation. Women with pregnancy complications were more likely to present to one of the health-care centres associated with each of the parent studies; therefore, attrition bias might have resulted in an overestimation of the occurrence of adverse outcomes. Third, three of the studies (MBOTE-SK, PREGMPOX, and Uvira mpox) were conducted in areas of active conflict. These conditions led to shortages in testing reagents, difficulty in transporting samples, and precluded the testing of additional placental samples and the differentiation between MPXV subclades Ia and Ib. Fourth, although the sample size is large compared with previous reports, the number of reported events was still small. The precision of some RRs estimated in the univariable analysis was low and the sample size was insufficient to conduct a multivariable analysis to adjust for differences in risk between women with and without adverse outcomes and between study sites. Furthermore, because of the small number of participants per study site, we were unable to model clustering by study. Therefore, the observed associations should be interpreted cautiously. Larger registries are needed to more accurately estimate the prevalence of adverse pregnancy outcomes linked to mpox and to establish the associated risk factors.

Pregnant women diagnosed with mpox should receive special clinical care, including counselling and psychological support throughout pregnancy and after delivery, particularly in cases of fetal loss or neonatal infection. For affected live neonates, long-term follow-up should be foreseen. There is also an urgent need to develop effective antiviral therapies that can be safely administered during pregnancy. Additionally, mpox prevention efforts, including vaccination, should prioritise pregnant women and key populations, such as adolescent girls, young women, and sex workers. Given that orthopoxviruses readily cross the placenta, first-generation and second-generation replicating vaccines based on vaccinia virus should be avoided during pregnancy. The third-generation, non-replicating MVA-BN vaccine showed no teratogenicity in preclinical studies and seems to be a safer alternative than replicating vaccine types; however, data on its safety in pregnant women remain scarce (<300 pregnancies).^28^ A phase 3 trial on the maternal safety and immunogenicity of this vaccine is ongoing in DR Congo (NCT06844500).

In summary, MPXV clade I infection during pregnancy has a high risk of adverse outcomes, including fetal loss and congenital infection, particularly when infection occurs in the first trimester. Targeted preventive and clinical strategies are urgently needed to protect pregnant women and their infants in endemic and epidemic settings of mpox.

### Contributors

### Data sharing

De-identified participant data will be made available from the corresponding author upon reasonable request—ie, when ethically viable without violating the protection of participants or other valid ethical, privacy, or security concerns.

## Declaration of interests

LL reports receiving institutional consultancy fees from BioNtech and institutional research funding from GSK, both of which were not relevant for this work. JK reports providing expert witness reports for the Treasury Board of Canada, and the Governments of Canada, Manitoba, and Alberta, none of which were relevant to this work. JK also reports receiving mpox research funding from the Canadian Institutes of Health Research and the Canada Research Chairs Program*.* All other authors declare no competing interests.
