# Supplementary material for: Maternal and neonatal outcomes after infection with monkeypox virus clade I during pregnancy in DR Congo: a pooled, prospective cohort study
Source: Lancet. 2026 Jan 17;407(10525):256–66. doi: 10.1016/S0140-6736(25)02309-8 (PMC12823291; doi:10.1016/S0140-6736(25)02309-8)
Supplement: Supplementary appendix [file mmc1.pdf]

THE LANCET

Supplementary appendix

This appendix formed part of the original submission and has been peer reviewed. We post it as supplied by the authors.

Supplement to: Vakaniaki EH, Barhishindi I, Mubiala A, et al. Maternal and neonatal outcomes after infection with monkeypox virus clade I during pregnancy in DR Congo: a pooled, prospective cohort study. *Lancet* 2025; published online Dec 19. https://doi.org/10.1016/S0140-6736(25)02309-8.

Supplementary appendix

Consortia author list 2
Supplementary Figure 1: Participant flow diagram 7
Supplementary Table 1: Baseline characteristics of participants with and without pregnancy outcome data 8
Supplementary Table 2: Pregnancy history and outcomes in pregnant women per trimester at the time of mpox diagnosis 10
Supplementary Table 3: Adverse pregnancy outcomes, according to clade 12
Supplementary Table 4: Pregnancy outcomes by original study 13
Statistical Analysis Plan 14

CONSORTIA AUTHOR LIST

The MBOTE-SK Consortium

Co-authors of the article. Affiliations listed in the article.
Emmanuel Hasivirwe Vakaniaki, Lydia Braunack-Mayer, Isabel Brosius, Stefanie Bracke, Eugene Bangwen, Elise De Vos, Sabin Sabiti Nundu, Daniel Mukadi-Bamuleka, Anne W. Rimoin, Tony Wawina-Bokalanga, Koen Vercauteren, Jason Kindrachuck, Nicola Low, Placide Mbala-Kingebeni, Laurens Liesenborghs

Other consortium members

Institut National de Recherche Biomédicale, Kinshasa, Democratic Republic of the Congo

- Prof. Steve Ahuka-Mundeke, Ph.D.
- Adrienne Amuri-Aziza, M.Sc.
- Jean-Claude Makangara-Cigolo, M.D.
- Fiston Mpinda Isekusu, M.Sc.
- Eddy Kinganda-Lusamaki, Ph.D.
- Raphaël Lumembe-Numbi, M.D.
- Yves Mujula, B.Sc.
- Papy Munganga Munganga, M.D.
- Jean-Claude Tshomba, B.Sc.

Department of Clinical Sciences, Institute of Tropical Medicine, Antwerp, Belgium

- Sarah Houben, Ph.D.
- Christophe Van Dijck, Ph.D.

Department of Epidemiology, Jonathan and Karin Fielding School of Public Health, University of California, Los Angeles, CA, USA

- Nicole A. Hoff, Ph.D.
- Sydney Merritt, M.P.H.

Département de Biologie Médicale, Cliniques Universitaires de Kinshasa, Université de Kinshasa, Kinshasa, Democratic Republic of the Congo

- Prof. Steve Ahuka-Mundeke, Ph.D.
- Eddy Kinganda-Lusamaki, Ph.D.
- Raphaël Lumembe-Numbi, M.D.

Institute of Social and Preventive Medicine, University of Bern, Bern, Switzerland and Graduate School of Cellular and Biomedical Sciences, University of Bern, Switzerland

- Jean-Claude Makangara-Cigolo, M.D.

TransVIHMI (Université de Montpellier, IRD, INSERM), Montpellier, France

- Martine Peeters, Ph.D.
- Eddy Kinganda-Lusamaki, Ph.D.

The Alliance for International Medical Action, Goma, Democratic Republic of the Congo

- Richard Kojan, M.D.

Kamituga Health Zone, South Kivu, Democratic Republic of the Congo

- Léandre Mutimbwa-Mambo, M.D.

Kamituga General Hospital, South Kivu, Democratic Republic of the Congo

- Jenestin Babingwa Munga, M.D.
- Steeven Bilembo Kitwanda, M.D.
- Divin Mazambi Mambo, M.D.
- Franklin Mweshi Kumbana, MD
- James Wakilongo Zangilwa, M.D.

National Program for Monkeypox and Viral Hemorrhagic Fevers, Mpox Incident Management System, Ministry of Health, Kinshasa, Democratic Republic of the Congo

- Cris Kacita, M.D.

The PREGMPOX consortium

Co-authors of the article. Affiliations listed in the article.

Isaac Barhishindi, Robert Colebunders, Christian Tshongo, Misaki Wayengera, Bruce Kirenga, David Lupande Mwenebitu, Joseph-Nelson Siewe-Fodjo, Susanne Krasemann, Patrick D. M. C. Katoto

Other consortium members

Center for Tropical Diseases and Global Health, Catholic University of Bukavu, Democratic Republic of the Congo

- Esto Bahizire, Ph.D.
- Wyvine Bapolisi, Ph.D.
- Bertin C. Bisimwa, M.Sc.
- Arsene Daniel Nyalundja, M.D.

Institut Supérieur des Techniques Médicales (ISTM-Bukavu)

- Bertin C. Bisimwa, M.Sc

Centre de Recherche en Sciences Naturelles de Lwiro, South Kivu, Democratic Republic of the Congo

- Esto Bahizire, Ph.D.

Department of Clinical Sciences, Liverpool School of Tropical Medicine, Liverpool, UnitedKingdom

- Arsene Daniel Nyalundja, M.D.

Makerere Lung Institute, Makerere University College of Health Sciences, Mulago Hospital, Kampala, Uganda

- Mudarshiru Bbuye, M.P.H.
- Claudine Mukashyaka, M.Sc.

Global Health Institute, University of Antwerp, Antwerp, Belgium.

- Marina Saleeb, M.P.H.

Department of Gynaecologie, Antwerp Surgical Training, Anatomy and Research Centre, University of Antwerp

- Prof. Yves Jacquemyn, Ph.D.

Molecular Pathology Group, Laboratory of Cell Biology and Histology & Laboratory of Medical Microbiology, Faculty of Medicine, University of Antwerp

- Prof. Samir Kumar-Singh, Ph.D.

University Medical Center Hamburg-Eppendorf, Hamburg, Germany

- Osama A.A. Mohamed, M.Sc.

<u>The PALM007-Consortium</u>

Co-authors of the article. Affiliations listed in the article.

Ange Mubiala, Bruce Nganga, Mireille Ngale, Gabriel Kayembe, Ali Dilu, Celestin Tshimanga, Jean-Luc Biampata, Nsengi Ntamabyaliro, Daniel Mukadi-Bamuleka, Jean-Jacques Muyembe-Tamfum, Veronique Nussenblatt, Ian Crozier, Lori E. Dodd, Olivier Tshiani-Mbaya, Placide Mbala-Kingebeni

Other consortium members

Institut National de Recherche Biomédicale, Kinshasa, Democratic Republic of the Congo

- Rosine Ali, Ph.D.

- Michael Kombozi Basika, B.Sc.
- Esaie Kindombe, B.Sc.
- Mays Kisala, M.D.
- Augustin Ibanda, B.Sc.
- Baudouin Matiba Baudouin, B.N.S.
- Gael Mukendi, B.Sc.
- Patrick Mutombo, B.Sc.

Department of Pharmacology, Faculty of Medicine, University of Kinshasa, Democratic Republic of the Congo

- Tona Lutete, Ph.D.
- Mariano Lusakibanza, Ph.D.
- Yves Lula, M.D.

Department of Anesthesia and Intensive Care, Faculty of Medicine, University of Kinshasa, Democratic Republic of the Congo

- Patricia Kabuni, M.D.

Ministry of Hygiene and Public Health, Democratic Republic of the Congo

- Hippy Lonza, M.D.
- Sylvain Mulumba, M.D.
- Henri Mutomboh, M.D.
- Yves-Ninon Kumuamba, M.D.

General Reference Hospital of Kole, Sankuru, Democratic Republic of the Congo

- Jules Alonga, M.D.

General Reference Hospital of Tunda, Maniema, Democratic Republic of the Congo

- Claude Shosongo, M.D.

National Institute of Allergy and Infectious Diseases, National Institutes of Health, Bethesda, MD, USA

- Katherine Cone, M.S.W.

The Uvira Study Group

Co-authors of the article. Affiliations listed in the article.
Espoir Bwenge Malembaka, Patrick Musole Bugeme, Andrew S. Azman

Other consortium members

Uvira General Reference Hospital, Democratic Republic of the Congo

- Trust Faraja Mukika, M.D.
- Patrick Kazuba Bugale, M.D.
- Salomon Mashupe Shangula, M.D.

Center for Tropical Diseases and Global Health, Université Catholique de Bukavu, DRC; Department of Epidemiology, Johns Hopkins Bloomberg School of Public Health, Baltimore, USA

- Levi Bugwaja, M.D.

Médecins Sans Frontières, Uvira, Democratic Republic of the Congo; Médecins Sans Frontières, Amsterdam, The Netherlands

- Stephanie Ngai, M.P.H.

Department of Epidemiology, Johns Hopkins Bloomberg School of Public Health, Baltimore, MD, USA

- Jules Jackson, M.Sc.

Supplementary Figure 1: Participant flow diagram

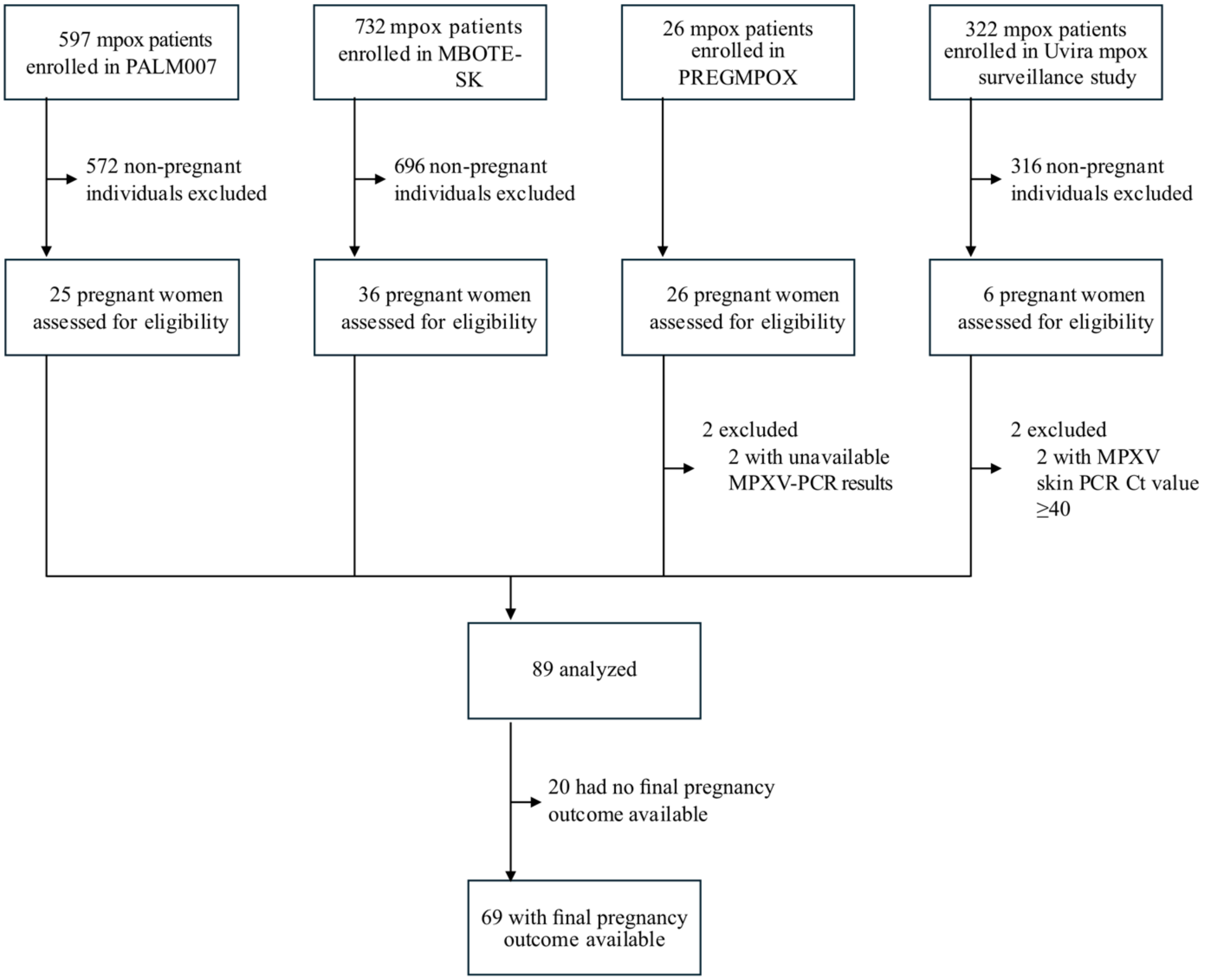

Supplementary Table 1: Baseline characteristics of participants with and without pregnancy outcome data

	Overall (N = 89)	Participantswit h un-known final pregnancy outcome (N = 20)	Participants with known final pregnancy outcome (N = 69)	p-value
**Recruitment setting**	..	..	..	0.004
Original study				
MBOTE-SK	36/89 (40%)	8/20 (40%)	28/69 (41%)	
PREGMPOX	24/89 (27%)	11/20 (55%)	13/69 (19%)	
PALM-007	25/89 (28%)	1/20 (5%)	24/69 (35%)	
Uvira mpox study	4/89 (5%)	0/20 (0%)	4/69 (6%)	
Province	..	..	..	0·012
South Kivu	64/89 (72%)	19/20 (95%)	45/69 (65%)	
Maniema	17/89 (19%)	0/20 (0%)	17/69 (25%)	
Sankuru	8/89 (9%)	1/20 (5%)	7/69 (10%)	
Outbreak setting*	..	..	..	0·01
Clade Ib/sh2023	64/89 (72%)	19/20 (95%)	45/69 (65%)	
Endemic Clade Ia	25/89 (28%)	1/20 (5%)	24/69 (35%)	
**Participant profile**	..	..	..	
Age, years	24 (20-30)	25 (19.75-30)	23 (20-30)	0·859
Age group	..	..	..	0·32
≤ 18 years	14/89 (16%)	4/20 (20%)	10/69 (15%)	
19-34 years	61/89 (69%)	15/20 (75%)	46/69 (67%)	
≥ 35 years	14/89 (16%)	1/20 (5%)	13/69 (19%)	
Occupation†	..	..	..	0·192
Home maker	35/89 (39%)	4/20 (20%)	31/69 (45%)	
Farmer	24/89 (27%)	9/20 (45%)	15/69 (22%)	
Businesswoman	18/89 (20%)	5/20 (25%)	13/69 (19%)	
Sex worker	4/89 (5%)	1/20 (5.0%)	3/69 (4%)	
Unemployed	4/89 (5%)	0/20 (0%)	4/69 (6%)	
Student	1/89 (1%)	0/20 (0%)	1/69 (1%)	
Other	3/89 (3%)	1/20 (5%)	2/69 (3%)	
**Exposure**	..	..	..	
Hunting in past 3 weeks	1/88 (1%)	0/20 (0%)	1/68 (1%)	>0·9
Consumed rodents in past 3 weeks	4/87 (5%)	0/20 (0%)	4/67 (6%)	0·6
Manipulated wildlife meat in past 3 weeks	10/86 (12%)	0/17 (0%)	10/69 (14%)	0·2
Consumption of wildlife in past 3 weeks	4/85 (5%)	0/20 (20%)	4/65 (6%)	0·569
Contact with other mpox case in past 3 weeks	57/73 (78%)	11/16 (69%)	46/57 (81%)	0·678

**Gestational trimester at the time of mpox diagnosis**	..	..	..	0·192
1st trimester (1-13 weeks gestational age)	25/89 (28%)	7/20 (35%)	18/69 (26%)	
2nd trimester (14-27 weeks gestational age)	31/89 (35%)	9/20 (45%)	22/69 (32%)	
3rd trimester (≥ 28 weeks gestational age)	33/89 (37%)	4/20 (20%)	29/69 (42%)	
**Obstetric history**	..	..	..	
Prior caesarean section	15/83 (18%)	2/19 (11%)	13/64 (20%)	0·50
Prior miscarriage	16/83 (19%)	4/20 (20%)	12/63 (19%)	1·00
Prior premature delivery	1/67 (2%)	1/15 (7%)	0/52 (0%)	0·349
Prior stillbirth	4/67 (6%)	2/15 (13%)	2/52 (4%)	0·371
**Attended prenatal consultation**	8/60 (13%)	2/9 (22%)	6/51 (12%)	0·04
**Signs**				
Number of lesions	62·0 (18·5-179·2)	68·5 (26·7-212·7)	60·0 (13-164·5)	0·391
WHO severity score	..	..	..	0·831
Mild (<25 lesions)	27/87 (31%)	5/20 (25%)	22/67 (33%)	
Moderate (25-99 lesions)	26/87 (30%)	7/20 (35%)	19/67 (28%)	
Severe (100-250 lesions)	20/87 (23%)	4/20 (20%)	16/67 (24%)	
Grave (>250 lesions)	14/87 (16%)	4/20 (20%)	10/67 (15%)	
**Clinical outcome during hospitalization**				
Duration of hospital stay, in days	13·0 (7·0-15·0)	6·0 (5·0-6·0)	13·0 (10·7-16·2)	<0·001

Data are n/N (%) for categorical variables or median (IQR) for numeric variables. N is the denominator representing the number of participants with available data. *Genomic sequencing analyses have revealed clade Ib/sh2023 or zoonotic clade Ia, no co-circulation was reported in the study areas as of May 2025. †Recorded as the main occupation of participant.
MBOTE-SK = Monkeypox Biology, Outcome, Transmission and Epidemiology - South Kivu, PREGMPOX= Pregnancy-Mpox. PALM-007= Pamoja Tulinde Maisha-007.

Supplementary Table 2: Pregnancy history and outcomes in pregnant women per trimester at the time of mpox diagnosis

	Pregnant mpox cases (N = 89)	Gestational trimester at the time of mpox diagnosis		
		1st trimester (1-13 weeks, N = 25)	2nd trimester (14-27 weeks, N = 31)	3rd trimester (≥ 28 weeks, N = 33)
**Obstetric history**	..	..	..	..
Female genital mutilation	0/84 (0%)	0/23 (0%)	0/30 (0%)	0/31 (0%)
Miscarriage	16/83 (19%)	3/23 (13%)	7/30 (23%)	6/30 (20%)
Preterm delivery	1/67 (2%)	0/16 (0%)	1/25 (4%)	0/26 (0%)
Stillbirth	4/67 (6%)	1/15 (7%)	2/25 (8%)	1/27 (4%)
Congenital anomalies	0/67 (0%)	0/16 (0%)	0/25 (0%)	0/26 (0%)
**Attended prenatal consultation**	8/60 (13%)	0/19 (0%)	3/22 (14%)	5/19 (26%)
**Complications documented during current pregnancy***	..	..	..	..
Gestational diabetes	0/60 (0%)	0/19 (0%)	0/21 (0%)	0/20 (0%)
Hypertension	0/79 (0%)	0/23 (0%)	0/27 (0%)	0/29 (0%)
Polyhydramnios	3/57 (5%)	0/12 (0%)	0/21 (0%)	3/24 (13%)
Preterm prelabor rupture of membranes	4/57 (7%)	1/13 (8%)	0/21 (0%)	3/23 (13%)
**Pregnancy complications during admission for mpox**	18/89 (20%)	11/25 (44%)	6/31 (19%)	1/33 (3%)
Abortion	11/18 (61%)	9/11 (82%)	2/6 (33%)	0/1 (0%)
Missed abortion	3/18 (17%)	1/11 (9%)	2/6 (33%)	0/1 (0%)
Stillbirth	3/18 (17%)	1/11 (9%)	1/6 (17%)	1/1 (100%)
Vaginal bleeding and abdominal pain without pregnancy loss	1/18 (6%)	0/11 (0%)	1/6 (17%)	0/1 (0%)
**Pregnancy outcomes**	..	..	..	..
Outcome data available	69/89 (78%)	18/25 (72%)	22/31 (71%)	29/33 (88%)
Delivery of healthy newborn	34/69 (49%)	1/18 (6%)	9/22 (41%)	24/29 (83%)
Adverse pregnancy outcome	35/69 (51%)	17/18 (94%)	13/22 (59%)	5/29 (17%)
Spontaneous abortion (<20 weeks)	16/69 (23%)	14/18 (78%)	2/22 (9%)	0/29 (0%)
Missed abortion (<20 weeks)	4/69 (6%)	1/18 (6%)	3/22 (14%)	0/29 (0%)
Stillbirth (>20 weeks)	11/69 (16%)	2/18 (11%)	8/22 (36%)	1/29 (3%)
Live preterm birth (<37 weeks)	0/69 (0%)	0/25 (0%)	0/31 (0%)	0/33 (0%)
Live newborn with macroscopic lesions	3/89 (3%)	0/25 (0%)	0/31 (0%)	3/33 (9%)
Live newborn with macroscopic lesions and neonatal death	1/89 (1%)	0/25 (0%)	0/31 (0%)	1/33 (3%)
Maternal death	0/69 (0%)	0/25 (0%)	0/31 (0%)	0/33 (0%)
Congenital anomalies	0/69 (0%)	0/25 (0%)	0/31 (0%)	0/33 (0%)
**MPXV-PCR-positive placental swab**	9/12 (75%)	2/2 (100%)	4/5 (80%)	3/5 (60%)

Ct value placental swab	23.6 (20.9, 32.2)	18.8 (18.1, 19.5)	23.4 (22.2, 25.7)	34.2 (30.1, 38)
**Duration from admission to adverse outcome, in days**	..	..	..	..
Spontaneous abortion (<20 weeks)	11.0 (6.8, 14.0)	11.0 (7.5, 14.0)	9.0 (7.5, 10.5)	..
Missed abortion (<20 weeks)	20.5 (18.8, 22.5)	21.0 (21.0, 21.0)	20.0 (17.5, 23.5)	..
Stillbirth (>20 weeks)	14.0 (12.5, 15.0)	..	13.5 (12.2, 14.0)	50.0 (50.0, 50.0)

Data are n/N (%) for categorical variables or median (IQR) for numeric variables. N is the denominator representing the number of participants with available data. *Any complications diagnosed during pregnancy. †Pregnancy complication occurring before discharge from the mpox treatment center. Ct=Cycle threshold. IQR=interquartile range. MPXV=monkeypox virus. PCR=polymerase chain reaction.

Supplementary Table 3: Adverse pregnancy outcomes, according to clade

	Pregnant mpox cases (N = 89)	Setting	
		Participants from Clade Ia affected areas (N = 25)	Participants from Clade Ib/sh2023 affected areas (N = 64)
**Pregnancy complications during admission for mpox**	18/89 (20%)	7/25 (28%)	11/64 (17%)
Spontaneous abortion (<20 weeks)	11/18 (17%)	5/7 (71%)	6/11 (55%)
Missed abortion (<20 weeks)	3/18 (17%)	2/7 (29%)	3/11 (27%)
Stillbirth (>20 weeks)	3/18 (17%)	0/7 (0%)	1/11 (9%)
Vaginal bleeding and abdominal pain without pregnancy loss	1/18 (6%)	0/7 (0%)	1/11 (9%)
**Pregnancy outcomes**	..	..	..
Outcome data available	69/89 (78%)	24/25 (96%)	45/64 (70%)
Delivery of healthy newborn	34/69 (49%)	11/24 (46%)	23/45 (51%)
Adverse pregnancy outcome	35/69 (51%)	13/24 (54%)	22/45 (49%)
Spontaneous abortion (<20 weeks)	16/69 (23%)	7/24 (29%)	9/45 (20%)
Missed abortion (<20 weeks)	4/69 (6%)	2/24 (8%)	2/45 (4%)
Stillbirth (>20 weeks)	11/69 (16%)	2/24 (8%)	9/45 (20%)
Live preterm birth (<37 weeks)	0/69 (0%)	0/24 (0%)	0/45 (0%)
Live term newborn with macroscopic lesions	3/89 (5%)	2/24 (8%)	1/64 (2%)
Live term newborn with macroscopic lesions and neonatal death	1/89 (1%)	0/24 (0%)	1/64 (2%)
Maternal death	0/69 (0%)	0/24 (0%)	0/45 (0%)
Congenital anomalies	0/69 (0%)	0/24 (0%)	0/45 (0%)

Data are n/N (%) for categorical variables or median (IQR) for numeric variables. N is the denominator representing the number of participants with available data. *Any complications diagnosed during pregnancy. †Pregnancy complication occurring before discharge from the mpox treatment center. Ct=Cycle threshold. IQR=interquartile range. MPXV=monkeypox virus. PCR=polymerase chain reaction.

Supplementary Table 4: Pregnancy outcomes by original study

	Overall (n=69)	Healthy newborn (n=34)	Adverse outcome (n=35)
Original study	..	..	..
MBOTE-SK	28	10/28 (36%)	18/28(64%)
PREGMPOX	13	10/13 (77%)	3/13 (23%)
PALM-007	24	11/24 (46%)	13/24 (54%)
Uvira mpox study	4	3/4 (75%)	1/4 (25%)

Data are all categorial variables n/N (%). ∗

Statistical Analysis Plan (SAP)

Assessment of mpox infection during pregnancy: a prospective cohort study of maternal and neonatal outcomes in the Democratic Republic of the Congo

SAP version 1.1, 28th August 2025

Table of Contents

Contributors 17

Revision history 18

Background 19

Eligibility criteria 20

Objectives and outcomes 20

- PRIMARY OBJECTIVES 20
- SECONDARY OBJECTIVES 26

Data management 29

Sample size considerations 29

Methodology 29

- DATA ANALYSIS SETS 29
- GENERAL PRINCIPLES FOR STATISTICAL METHODS 30
- MISSING VALUES 30
- STATISTICAL SOFTWARE 31
- PLANNED SUBGROUP AND SENSITIVITY ANALYSES 31

References 32

CONTRIBUTORS

Name	Affiliation	Role in SAP writing
Dr Lydia Braunack-Mayer	Institute of Social and Preventive Medicine, University of Bern	Author
Ange Mubiala	Institut National de Recherche Biomédicale	Author
Dr Emmanuel Hasivirwe Vakaniaki	Institut National de Recherche Biomédicale	Author
Dr Bruce Nganga	Institut National de Recherche Biomédicale	Researcher
Dr Isaac Barhishindi	Hôpital Provincial Général de Référence de Bukavu	Researcher
Dr Stefanie Bracke	Institute of Tropical Medicine	Reviewer
Prof Nicola Low	Institute of Social and Preventive Medicine, University of Bern	Reviewer
Dr Olivier Mbaya-Tshiani	Frederick National Laboratory for Cancer Research	Reviewer
Dr Ian Crozier	Frederick National Laboratory for Cancer Research	Reviewer
Dr Veronique Nussenblatt	National Institute of Allergy and Infectious Diseases	Reviewer
Dr Laurens Liesenborghs	Institute of Tropical Medicine	Co-Principal Investigator MBOTE-SK
Prof Sabin Sabiti	Institut National de Recherche Biomédicale	Co-Principal Investigator MBOTE-SK
Prof Placide Mbala	Institut National de Recherche Biomédicale	Lead PALM007, Co-Principal Investigator MBOTE-SK
Prof Patrick Katoto	Université Catholique de Bukavu	Principal Investigator PREGMPOX
Dr Lori Dodd	National Institute of Allergy and Infectious Diseases	Lead PALM007
Dr Espoir Malembaka	Université Catholique de Bukavu	Co-Principal Investigator Uvira mpox study

REVISION HISTORY

Version	Section	Summary of changes
1.0	N/A	First version.
1.1	Primary objectives	In primary objective 4, corrected terminology 'Number of previous pregnancies' to 'Parity'.<br>In primary objective 6, infection with syphilis during hospitalization added to outcomes measured for primary objective 6.<br>In primary objective 7, replaced the categories for 'Description of pregnancy complications during hospitalization' with four distinct groups: spontaneous abortion, missed abortion, stillbirth, or vaginal bleeding and abdominal pain without pregnancy loss.<br>In primary objective 8, removed low birth weight from the list of potential adverse pregnancy outcomes, as data on birth weight was difficult to verify and all neonates identified as low birth weight were also identified as having another adverse pregnancy outcome.<br>In primary objective 8, positive oropharyngeal PCR swab removed from the definition of fetal or neonatal infection.
	Secondary objectives	In secondary objective 3, removed the requirement for a minimum set of variables to be adjusted for.<br>In secondary objective 3, maternal age, presence of fever, genital skin lesions and suspected clade differentiation at baseline added to list of the variables to be adjusted for.

BACKGROUND

In recent years, mpox, a zoonotic viral disease caused by the monkeypox virus (MPXV), has gained global attention and emerged as an international public health concern.[1] The disease affects individuals of all ages. However, with changing demography and epidemiology, and with increasing human-to-human transmission of MPXV, women of childbearing age, including pregnant women, have become increasingly affected.[2,3]

The epidemiology of mpox infection during pregnancy remains unclear. Limited clinical studies and case series, including small numbers of pregnant women, have reported adverse outcomes such as miscarriage, intrauterine fetal demise, preterm birth, and congenital mpox, supporting vertical transmission.[3,4] These adverse outcomes have been observed across all three trimesters. However, a detailed description of adverse outcomes, according to the pregnancy trimester in which the woman became infected, and of factors associated with them, are lacking.[5] Comprehensive prospective cohort studies are needed, particularly in regions where MPXV is endemic, as in the Democratic Republic of the Congo (DRC).[5,6]

To address this gap, the present study investigates the maternal, fetal and neonatal outcomes of MPXV infection during pregnancy through a multi-center, prospective cohort study in the DRC, aiming to better understand the impact of MPXV infection on pregnancy and fetal or neonatal outcomes. This cohort study combines records of pregnant women from four separate cohorts: Mpox Biology, Outcome, Transmission, and Epidemiology in South Kivu (MBOTE-SK), Pamoja Tulinde Maisha 007 (PALM007), Impact of MPXV Infection on Pregnancy Outcome and Newborn Health (PREGMPOX), and the Uvira mpox study. Together, these studies cover health zones in three provinces in the DRC: South Kivu, Sankuru and Maniema.

MBOTE-SK is a prospective observational cohort study focused on the clinical and virological characterization of MPXV infections in individuals infected with Clade Ib MPXV in the Kamituga health zone, South Kivu. All confirmed cases, including in pregnant women, are followed during hospitalization until discharge, with two follow-up visits scheduled on day 29 and day 59 (ClinicalTrials.gov: NCT06652646). Pregnant women are encouraged to return to the treatment center if they have any new or persistent symptoms or pregnancy complications, and for delivery. **PALM007** is a double-blind, randomized controlled trial comparing 14-days oral tecovirimat with placebo in patients with mpox. Eligibility criteria include individuals of any age or pregnancy status, with at least one skin lesion and MPXV confirmed by PCR from blood, oropharyngeal, or lesion samples (ClinicalTrials.gov: NCT05559099). The study was conducted at two general hospitals: Tunda hospital in Maniema province and Kole hospital in Sankuru province.
PREGMPOX is a prospective observational cohort study assessing the impact of MPXV infection on pregnancy outcomes and newborn health, aiming to improve understanding of the effects of MPXV during pregnancy and on pregnancy outcomes. This study is conducted in four health zones (Nyangezi, Kadutu, Miti-Murhesa, Nyantende) in the province of South Kivu.

Uvira mpox study is an mpox surveillance study in the Uvira health zone, including all suspected cases at the treatment center. Data collection included pregnancy, breastfeeding, and child nutrition, with targeted home visits for pregnant women.

ELIGIBILITY CRITERIA

Individuals were considered eligible for this study if they met the following inclusion criteria:

- Recruited into MBOTE-SK, PALM007, PREGMPOX or the Uvira mpox study.
- Pregnant at the time of hospitalization.
- MPXV confirmed by PCR from blood, oropharyngeal, or lesion samples, tested by PCR GeneXpert the Cepheid Xpert® Mpox assay on the GeneXpert system, targeting non-variola Orthopoxvirus and MPXV Clade II DNA or Radi PCR kit (KH Medical) which detects Clade I, Clade II MPXV, and generic Orthopoxvirus via specific fluorescence channels, with Ct <40.
- Individuals were excluded if they were not pregnant or tested negative to MPXV by PCR from blood, oropharyngeal, or lesion samples.

OBJECTIVES AND OUTCOMES

The general objectives of this analysis are to describe the impact of MPXV infection during pregnancy and to determine the frequency of adverse maternal and fetal or neonatal outcomes by trimester of pregnancy.

PRIMARY OBJECTIVES

1. To describe the socio-demographic factors of pregnant women infected with MPXV, according to the trimester of pregnancy.

We will summarize the socio-demographic characteristics of women at baseline, the time of their hospitalization for treatment of MPXV, both overall and stratified by the trimester of pregnancy. We will compare characteristics between trimesters of pregnancy.

Table 1: Specific outcomes measured for primary objective one.

Outcome	Variable used or derivation	Variable type	Statistic
Gestational age at baseline, in weeks	`‘Gestational age at the moment of hospitalization for mpox’`	Numeric	Median, IQR
Trimester of pregnancy at baseline	`‘Gestational age at the moment of hospitalization for mpox’ < 14; 14 <= ‘Gestational age at the moment of hospitalization for mpox’ < 28; 28 <= ‘Gestational age at the moment of hospitalization for mpox’`	Categorical	n, %
Province at baseline	`‘Province’`	Categorical	n, %
Health zone at baseline	`‘Health zone’`	Categorical	n, %
Age at baseline, in years	`‘Age (years)’`	Numeric	Median, IQR
Age category at baseline	`‘Age (years)’ <= 18; 18 < ‘Age (years)’ <= 34; 35 < ‘Age (years)’`	Categorical	n, %
Occupation at baseline	`‘Profession’`	Categorical	n, %

For all objectives that refer to gestational age or trimester of pregnancy at baseline, gestational age is estimated in weeks: by ultrasound when available, or clinically using the last menstrual period when ultrasound was not performed. Days are rounded up or down to report whole weeks. Trimesters of pregnancy are defined as: 1st (0 to 13 weeks + 6 days of gestation), 2nd (14 weeks to 27 weeks + 6 days), 3rd (28 weeks to delivery).[7]

2. **To describe the exposure of pregnant women infected with MPXV, according to the trimester of pregnancy.**

We will summarize the exposure characteristics of women at baseline, both overall and stratified by the trimester of pregnancy. We will compare characteristics between trimesters of pregnancy.

Table 2: Specific outcomes measured for primary objective two.

Outcome	Variable used or derivation	Variable type	Statistic
Participated in hunting	'Participated in hunting in last 3 weeks'	Binary	n, %
Consumed rodents	'Consumed rodents in last 3 weeks'	Binary	n, %
Manipulated bushmeat	'Manipulated bushmeat in last 3 weeks?'	Binary	n, %
Consumed bushmeat	'Bushmeat consumption in last 3 weeks'	Binary	n, %
Had contact with a suspected or confirmed mpox case	'Contact with suspected or confirmed mpox case in last 3 weeks'	Binary	n, %
If contact with a suspected or confirmed mpox case was made, relationship to this contact	'Relationship or contact with mpox case'	Categorical	n, %
If contact with a suspected or confirmed mpox case was made, type of contact	'Types of contact'	Categorical	n, %
Most likely source of infection	'Most likely source of infection'	Categorical	n, %

3. **To describe the medical history of pregnant women hospitalized with mpox by trimester of pregnancy.**

We will summarize the vaccination history and history of co-infections of women at baseline, both overall and stratified by the trimester of pregnancy. We will compare characteristics between trimesters of pregnancy.

Table 3: Specific outcomes measured for primary objective three.

Outcome	Variable used or derivation	Variable type	Statistic
Uses alcohol	'Alcohol use?'	Binary	n, %
Uses tobacco	'Tobacco use?'	Binary	n, %
Childhood history of smallpox vaccination	'Received smallpox vaccination during childhood?'	Binary	n, %
Receipt of mpox vaccination with at least one dose of the MVA-BN vaccine	'Received recent mpox vaccination with MVA-BN?'	Binary	n, %
Living with HIV at baseline	'Living with HIV?'	Binary	n, %
Living with active tuberculosis at baseline	'Active tuberculosis?'	Binary	n, %

Living with active renal disease at baseline	‘Active renal disease?’	Binary	n, %
Living with active liver disease at baseline	‘Active liver disease?’	Binary	n, %

In the DRC, HIV infection (living with HIV or tested positive to HIV RDT) is diagnosed using a national algorithm, which is based on three sequential rapid diagnostic tests:

1. Determine™ HIV-1/2 (screening test).
2. HIV 1/2 Stat-Pak® (confirmatory test).
3. Uni-Gold™ HIV (tie-breaker if the first two results are discordant).

4. To describe the obstetric history of pregnant women hospitalized with mpox by trimester of pregnancy.

We will summarize the obstetric history of women at baseline, both overall and stratified by the trimester of pregnancy. We will compare characteristics between trimesters of pregnancy.

Table 4: Specific outcomes measured for primary objective four.

Outcome	Variable used or derivation	Variable type	Statistic
Gestational age at baseline, in weeks	‘Gestational age at the moment of hospitalization for mpox’	Numeric	n, %
Trimester of pregnancy at baseline	‘Gestational age at the moment of hospitalization for mpox’ < 14; 14 <= ‘Gestational age at the moment of hospitalization for mpox’ < 28; 28 <= ‘Gestational age at the moment of hospitalization for mpox’	Categorical	Median, IQR
Attended prenatal consultations	‘# of prenatal consultations attended’	Binary	n, %
Presence of female genital mutilation	‘Female genital mutilation’	Binary	n, %
Parity	‘# of previous pregnancies’	Numeric	Median, IQR
Number of previous miscarriages	‘# of previous miscarriages’	Numeric	Median, IQR
Number of previous vaginal deliveries	‘# of previous vaginal deliveries’	Numeric	Median, IQR
Number of previous ceasarean sections	‘# of ceasarean section’	Numeric	Median, IQR
History of premature delivery	‘History of premature delivery’	Binary	n, %
History of stillbirth	‘History of stillbirth’	Binary	n, %
History of children with congenital anomalies	‘Children with congenital anomalies’	Binary	n, %

Gestational age or trimester of pregnancy at baseline are defined as in Primary objective one.

5. Describe the symptoms and signs characteristics of pregnant women infected with MPXV at inclusion.

We will summarize the clinical symptoms and signs of infection at baseline, both overall and stratified by the trimester of pregnancy. We will compare characteristics between trimesters of pregnancy.

Table 5: Specific outcomes measured for primary objective five.

**Outcome**	**Variable used or derivation**	**Variable type**	**Statistic**
Number of days between the onset of symptoms and hospitalization, in days	`‘# of days between symptom onset and inclusion’`	Numeric	Median, IQR
Symptoms			
Presence of fever at baseline	`‘Fever’`	Binary	n, %
Presence of fatigue at baseline	`‘Fatigue’`	Binary	n, %
Presence of itching at baseline	`‘Itching’`	Binary	n, %
Presence of myalgia or arthralgia at baseline	`‘Myalgia/arthralgia’`	Binary	n, %
Presence of pain in lesions at baseline	`‘Pain in lesions’`	Binary	n, %
Prescence of cough at baseline	`‘Cough’`	Binary	n, %
Presence of sore throat or dysphagia at baseline	`‘Sore throat/dysphagia’`	Binary	n, %
Presence of headache at baseline	`‘Headache’`	Binary	n, %
Presence of anorexia at baseline	`‘Anorexia’`	Binary	n, %
Presence of abdominal pain at baseline	`‘Abdominal pain’`	Binary	n, %
Presence of vomiting or nausea at baseline	`‘Vomiting/nausea’`	Binary	n, %
Presence of diarrhea at baseline	`‘Diarrhea’`	Binary	n, %
Presence of mictalgia (dysuria) at baseline	`‘Mictalgia’`	Binary	n, %
Presence of genital pain at baseline	`‘Genital pain’ = 1 OR ‘Vaginal pain’ = 1`	Binary	n, %
Presence of genital edema at baseline	`‘Genital edema’`	Binary	n, %
Presence of rectal pain at baseline	`‘Rectal pain’`	Binary	n, %
Presence of eye pain at baseline	`‘Eye pain’`	Binary	n, %
Presence of convulsions or altered consciousness at baseline	`‘Convulsions or altered consciousness’`	Binary	n, %
Signs			
Number of skin lesions at baseline	`‘# of skin lesions’`	Numeric	Median, IQR
Severity of infection at baseline	`‘# of skin lesions’ < 25; 25 <= ‘# of skin lesions’ < 100; 100 <= ‘# of skin lesions’ < 250; 250 <= ‘# of skin lesions’`	Categorical	n, %
Types of skin lesions present at baseline	`‘Types of skin lesions’`	Categorical	n, %
Presence of genital skin lesions at baseline	`‘Genital skin lesions’`	Binary	n, %
Presence of rectal lesions at baseline	`‘Rectal lesions’`	Binary	n, %
Presence of lymphadenopathy at baseline	`‘Lymphadenopathy?’`	Binary	n, %

Infection severity is defined from the number of skin lesions according to WHO severity score: mild (<25 lesions), moderate (25–99), severe (100–250) and grave (>250 lesions).[8]

6. **Describe co-infections during hospitalization among pregnant women infected with MPXV, by pregnancy trimester.**

We will summarize the presence of pregnancy complications and co-infections during hospitalization, both overall and stratified by the trimester of pregnancy. We will compare characteristics between trimesters of pregnancy.

Table 6: Specific outcomes measured for primary objective six.

Outcome	Variable used or derivation	Variable type	Statistic
Infection with acute malaria during hospitalization	`‘Acute malaria during hospitalization?’`	Binary	n, %
Infection with syphilis during hospitalization	`‘Syphillis’`	Binary	n, %
Other acute co-infections during hospitalization	`‘Other acute co-infections during hospitalization’`	Categorical	n, %

Acute malaria during hospitalization is detected by rapid diagnostic test or thick blood smear. Syphilis is detected by a Rapid Plasma Reagin (RPR) non-treponemal blood test.

7. **Describe the clinical outcomes of pregnant women during hospitalization, according to the trimester of pregnancy.**

We will summarize the hospitalization outcomes of women, both overall and stratified by the trimester of pregnancy. We will compare characteristics between trimesters of pregnancy.

Table 7: Specific outcome measures for primary objective seven.

Outcome	Variable used or derivation	Variable type	Statistic
Duration of hospital stay, in days	`‘Date discharged from hospital’ – ‘Inclusion date’`	Numeric	Median, IQR
Time from symptom onset to hospital discharge, in days	`‘# days between symptom onset and inclusion’ + ‘Date discharged from hospital’ – ‘Inclusion date’`	Numeric	Median, IQR
Death during hospitalization	`‘Discharged dead or alive’`	Binary	n, %
Duration of rash from admission, in days	`‘Duration of rash (days) from admission to scab falling off’`	Numeric	Median, IQR
Receipt of tecovirimat during hospitalization	`‘Received tecovirimat’`	Binary	n, %
Pregnancy complications during acute hospitalization	`‘Pregnancy complications during acute hospitalization?’`	Binary	n, %
Description of pregnancy complications during hospitalization	`Categorized from free text entries to ‘If yes, describe complications’: spontaneous abortion; missed abortion; stillbirth; vaginal bleeding and abdominal pain without pregnancy loss`	Categorical	n, %

8. **Describe pregnancy outcome among pregnant women with MPXV, according to the trimester of admission**.

Among women for whom a birth record is available, we will summarize the pregnancy outcomes of women hospitalized with MPXV, both overall and stratified by the trimester of pregnancy. We will compare characteristics between trimesters of pregnancy.

Table 8: Specific outcome measures for primary objective eight.

Outcome	Variable used or derivation	Variable type	Statistic

Gestational age at time of delivery, in weeks	`‘Gestational age at time of delivery’`	Numeric	Median, IQR
Maternal death	`‘Maternal death’`	Binary	n, %
Spontaneous abortion	`‘Pregnancy outcome’ = “spontaneous abortion (<20 weeks)” OR “missed abortion (<20 weeks)”`	Binary	n, %
Stillbirth	`‘Pregnancy outcome’ = “stillbirth (≥20 weeks)”`	Binary	n, %
Preterm live birth	`‘Pregnancy outcome’ = “preterm alive birth”`	Binary	n, %
Presence of macroscopic lesions on the fetus or neonate	`‘Mascroscopic lesions on fetus or newborn’`	Binary	n, %
Positive placental PCR swab	`‘PCR swab placenta’`	Binary	n, %

We will also define a composite outcome, any adverse pregnancy, fetal or neonate outcome, to include any pregnancy-related complication that is potentially linked to the MPXV infection. Recognized or suspected adverse outcomes are as follows.[9]

- **Birth outcomes:**
  - spontaneous abortion (pregnancy loss before 20 completed weeks of gestation)
  - stillbirth (fetal death at or after 20 completed weeks of gestation)
  - preterm birth (live birth before 37 completed weeks of gestation)
- **Fetal or neonate outcomes:**
  - fetal or neonatal infection (presence of macroscopic skin lesions
- **Maternal outcomes:**
  - maternal death

The presence of macroscopic skin lesions is defined as: macule, papule, vesicle, small pustule, umbilical pustule, ulcerated lesion, crust, scar. PCR testing is performed with the Cepheid Xpert® Mpox assay on the GeneXpert system, targeting non-variola Orthopoxvirus and MPXV Clade II DNA or Radi PCR kit (KH Medical) which detects Clade I, Clade II MPXV, and generic Orthopoxvirus via specific fluorescence channels, with Ct <40.

9. Describe post-partum obstetrical characteristics of women hospitalized with MPXV.

Among women for whom a birth record is available, we will summarize the post-partum obstetrical characteristics of women hospitalized with MPXV, both overall and stratified by the trimester of pregnancy. We will compare characteristics between trimesters of pregnancy.

Table 9: Specific outcome measures for primary objective nine.

Outcome	Variable used or derivation	Variable type	Statistic
Type of delivery (caesarean or vaginal)	`‘Type of delivery’`	Categorical	n, %
Among caesarean deliveries, indication for caesarean delivery	`Categorized from free-text entries to ‘Indication for caesarean section’: history of caesarean section, prolonged labor, abnormal positioning`	Categorical	n, %
Weight of the fetus or neonate, in grams	`‘Weight newborn’`	Numeric	Median, IQR

SECONDARY OBJECTIVES

1. **To describe the presence of MPXV-DNA in various samples with Ct value collected at diagnosis from pregnant women, stratified by trimester.**

We will describe the MPXV PCR results of samples taken at the time of hospitalization, both overall and stratified by the trimester of pregnancy. We will compare results between trimesters of pregnancy.

Table 10: Specific outcome measures for secondary objective one.

Outcome	Variable used or derivation	Variable type	Statistic
Presence of MPXV-DNA in skin lesion samples collected at baseline	`‘PCR skin lesion’`	Binary	n, %
Ct value for MPXV PCR on skin lesions at baseline	`‘CT value skin lesion’`	Numeric	Median, IQR
Presence of MPXV-DNA in oropharyngeal swab samples collected at baseline	`‘PCR oropharyngeal swab’`	Binary	n, %
Ct value for MPXV PCR on oropharyngeal swabs at baseline	`‘CT value oropharyngeal swab’`	Numeric	Median, IQR
Presence of MPXV-DNA in blood taken at baseline	`‘PCR blood’`	Binary	n, %
Ct value for MPXV PCR on blood at baseline	`‘CT value blood’`	Numeric	Median, IQR

2. **To describe the presence of MPXV-DNA in outcomes samples collected at the delivery or following an adverse pregnancy outcome.**

Among women with a birth record available, we will describe the MPXV PCR results of samples taken from both mother and neonate or fetus at the time of delivery, both overall and stratified by the trimester of pregnancy. We will compare results between trimesters of pregnancy.

Table 11: Specific outcome measures for secondary objective two.

Outcome	Variable used or derivation	Variable type	Statistic
Presence of MPXV-DNA in skin lesion samples collected from the mother at delivery	`‘PCR skin lesions mother’`	Binary	n, %
Ct value for MPXV PCR on skin lesions from the mother at delivery	`‘CT value skin lesions mother’`	Numeric	Median, IQR
Presence of MPXV-DNA in vaginal swabs collected from the mother at delivery	`‘PCR vaginal swab mother’`	Binary	n, %
Ct value for MPXV PCR on vaginal swabs from the mother at delivery	`‘CT value vaginal swab mother’`	Numeric	Median, IQR
Presence of MPXV-DNA in breastmilk samples collected from the mother at delivery	`‘PCR mother milk’`	Binary	n, %
Ct value for MPXV PCR on breastmilk from the mother at delivery	`‘CT value mother milk’`	Numeric	Median, IQR

Presence of MPXV-DNA in oropharyngeal swabs collected from the mother at delivery	`‘PCR oropharyngeal swab mother’`	Binary	n, %
Ct value for MPXV PCR on oropharyngeal swabs from the mother at delivery	`‘CT value oropharyngeal swab mother’`	Numeric	Median, IQR
Presence of MPXV-DNA in blood collected from the mother at delivery	`‘PCR blood mother’`	Binary	n, %
Ct value for MPXV PCR on blood collected from the mother at delivery	`‘CT value blood mother’`	Numeric	Median, IQR
Presence of MPXV-DNA in oropharyngeal swabs collected from the neonate or fetus at delivery	`‘PCR oropharyngeal swab fetus or newborn’`	Binary	n, %
Ct value for MPXV PCR on oropharyngeal swabs from the neonate or fetus at delivery	`‘CT value swab fetus or newborn’`	Numeric	Median, IQR
Presence of MPXV-DNA in abortion products used at delivery	`‘PCR swab abortion products’`	Binary	n, %
Ct value for MPXV PCR on abortion products used at delivery	`‘CT value swab abortion products’`	Numeric	Median, IQR
Presence of MPXV-DNA in placental swabs collected at delivery	`‘PCR swab placenta’`	Binary	n, %
Ct value for MPXV PCR on placental swabs collected at delivery	`‘CT value swab placenta’`	Numeric	Median, IQR

Abortion products refer to the tissues expelled following an abortion, including embryonic or fetal tissue, the gestational sac, placental tissue, amniotic membranes, and decidua.

3. Identify factors associated with adverse pregnancy outcomes.

Among women with a birth record available, we will evaluate associations between risk factors for adverse birth outcomes and any adverse pregnancy, fetal or neonate outcome among women hospitalized for MPXV. We will do this by, first, summarizing counts (with %) or medians (with IQR) for women who did or did not have an adverse outcome.

We will also report associations between risk factors and adverse birth outcomes as risk ratios. Statistical methods and principles for this analysis are described in detail in the section **General principles for statistical methods**. In brief, if sample size allows, Poisson regression will be used to estimate adjusted risk ratios for associations between risk factors and adverse outcomes. To avoid collinearity, the variables “Severity of infection at baseline” and “Low Ct value or high viral load at baseline” will not be included in the same model.

Table 12: Specific outcome measures and risk factors for secondary objective three.

Concept	Variable used or derivation	Variable type	Statistic
Outcomes			
Any adverse pregnancy, fetal or neonate outcome	`Defined as part of Primary Objective 8`	Binary	n, %, RR
Risk factors, in order of their a-prior strength of association			
Maternal age at baseline	`‘Age (years)’`	Continuous	Median, IQR
Trimester of pregnancy at baseline	`‘Gestational age at the moment of hospitalization for`	Categorical	n, %, RR

	mpox' <= 12; 12 < 'Gestational age at the moment of hospitalization for mpox' <= 26; 26 < 'Gestational age at the moment of hospitalization for mpox'		
Number of skin lesions at baseline	'# of skin lesion'	Continuous	Median, IQR
Severity of infection at baseline	100 <= '# of skin lesions'; '# of skin lesions' < 100	Binary	n, %, RR
Ct value on skin lesions at baseline	'CT value skin lesion'	Continuous	Median, IQR
Ct value on oropharyngeal swabs at baseline	'CT value oropharyngeal swab'	Continuous	Median, IQR
Low Ct value or high viral load at baseline from PCR on skin lesions or oropharyngeal swabs, at baseline	'CT value skin lesion' <= 30 OR, if 'CT value skin lesion' is NA, 'CT value oropharyngeal swab' <= 30; 30 < 'CT value skin lesion' OR NA, 'CT value oropharyngeal swab'	Binary	n, %, RR
If contact with a suspected or confirmed mpox case was made, type of contact	'Types of contact'	Categorical	n, %, RR
Living with HIV at baseline	'Living with HIV?'	Binary	n, %, RR
Fever at baseline	'Fever'	Binary	n, %, RR
Infection with acute malaria during hospitalization	'Acute malaria during hospitalization?'	Binary	n, %, RR
Other acute co-infections during hospitalization	'Other acute co-infections during hospitalization'	Categorical	n, %, RR
Presence of genital lesions at baseline	'Genital skin lesions'	Binary	n, %, RR
Suspected clade differentiation based on health zone	'Health zone'	Categorical	n, %, RR

Any adverse pregnancy, fetal or neonate outcome is defined as in Primary Objective eight. Trimester of pregnancy is defined as in Primary Objective one. Acute malaria during hospitalization is detected by rapid diagnostic test or thick blood smear.

4. **Describe maternal and neonatal complications, sequelae, and outcomes after delivery.**

Among women with a birth record available, we will summarize maternal and neonatal complications, sequelae, and outcomes after delivery.

Table 13: Specific outcome measures for secondary objective four.

Concept	Variable used or derivation	Variable type	Statistic
Occurrence of a postnatal visit to the mother	'Follow-up postnatal mother visit'	Binary	n, %
Number of days after delivery when the postnatal visit to the mother occurred	'If yes, how many days after delivery'	Numeric	Median, IQR
Occurrence of persistent symptoms, complications or sequelae after delivery	Categorized from free text entries to 'List any persistent symptoms, complications, or sequelae'	Categorical	n, %
Occurrence of a postnatal visit to the neonate	'Follow-up postnatal infant visit'	Binary	Median, IQR

Number of days after delivery when the postnatal visit to the neonate occurred	'If yes, how many days after birth'	Numeric	n, %
Occurrence of persistent symptoms, complications or sequelae after delivery	Categorized from free text entries to 'List any persistent symptoms, complications, or sequelae, outcome'	Categorical	n, %
Cause of neonate death	Categorized from free text entries to 'If death, cause of death'	Categorical	n, %
If death occurred, time from delivery to death, in days	Number extracted from free text entries to 'Time from birth to death in days'	Numeric	Median, IQR

DATA MANAGEMENT

Data are entered into a Microsoft Excel spreadsheet. A Microsoft Excel spreadsheet will be provided for statistical analyses.

SAMPLE SIZE CONSIDERATIONS

This study was designed to generate preliminary estimates of the prevalence of mpox-related complications during pregnancy and to assess the potential impact of MPXV infection on maternal and neonatal outcomes. Given the need for data in the context of ongoing MPXV transmission, no formal sample size calculation was performed. Instead, pregnant women were identified through recruitment into ongoing cohort studies in the DRC. We expect to include approximately 90 pregnant women infected with MPXV, with birth outcomes available for approximately 60 (67%) of women.

All statistical analyses are planned with this small sample size in mind. Proportions will be reported with exact confidence intervals and hypothesis testing will be performed with exact methods. If regression analyses are feasible, they will be conducted with methods suitable for small sample sizes and sparse data. We acknowledge that regression models may not converge or produce stable estimates, and that it may only be possible to report descriptive and bivariate analyses. As a result, care will be taken in the interpretation and communication of findings. We will present all estimates with measures of uncertainty, and will qualify conclusions to reflect the exploratory nature of this study.

METHODOLOGY

DATA ANALYSIS SETS

We will perform analyses on two different sets of individuals:

- **Full analysis set:** All women included in the cohort study, regardless of whether a birth record is available. This analysis set will be used to perform analyses related to Primary Objectives one to seven and Secondary Objective one.
- **Births analysis set:** A subset of women with birth records. This analysis set will be used to perform analyses related to Primary Objectives eight and nine, and Secondary Objectives two, three and four, and will exclude women for whom a birth record is not available.

The unit of analysis is the pregnant woman. In case a woman is enrolled more than once with separate pregnancies, each pregnancy will be analysed as a separate unit.

GENERAL PRINCIPLES FOR STATISTICAL METHODS

Analysis of primary and secondary objectives

In general, categorical outcomes will be reported as counts and percentages (%), reported with their corresponding Clopper Pearson confidence intervals. Continuous variables will be summarized with the median, interquartile range and total range. All statistics will be reported both for all women and for women in each trimester of pregnancy. Proportions for categorical outcomes will be compared between trimesters with Fisher's exact test. Medians for continuous variables will be compared between trimesters with the Kruskal-Wallis test. P-values will be reported but will be interpreted cautiously given the small sample size and exploratory nature of the study.

Analysis of risk factors

Risk factors are specified a-priori based on existing evidence and subject-matter expertise. Given our small sample size and potential for sparse data, our analysis of risk factors may be limited to reporting crude, unadjusted estimates. Risk ratios will be calculated for the relationship between exposure to each binary or categorical risk factor and outcomes variables. To calculate risk ratios for continuous variables, binary or categorical variables will be derived from continuous risk factors. 95% confidence intervals will be computed using bootstrap confidence intervals. Risk ratios will be reported both for all women and for women in each trimester of pregnancy, but we do not plan to perform statistical hypothesis testing to compare between trimesters.

We will attempt to use multivariable regression models to calculate adjusted risk ratios. Separate models will be fitted for each outcome, with the outcome as the dependent variable and risk factors as exposure variables. Confounder variables will be specified a-priori based on existing evidence and subject-matter expertise.

Regression analyses will be conducted using Poisson regression. Risk ratios will be reported with their corresponding 95% confidence intervals. Robust standard errors or bootstrap confidence intervals will be used in the case of over- or under-dispersion. We will assess model performance descriptively, although we recognize that traditional goodness-of-fit metrics may not be reliable in small samples. We acknowledge that regression models may not converge or produce stable estimates. If this occurs, we will limit the selection of risk factors, defined a-priori, and will not adjust for confounding. Otherwise, only unadjusted descriptive and bivariate analyses will be reported.

MISSING VALUES

We will report the number and percentage (%) of missing data for all variables. Given the small sample size, we will not impute missing observations and analyses will be performed with complete cases. Variables with large amounts of missing data (for example, more than 20%) will be excluded from regression-based analyses.

Statistical software

Analyses will be performed with R version 4.3.0 and SPSS version 27.0, or more recent. AI-based tools, such as GitHub Copilot, may be used to support code development.

Planned subgroup and sensitivity analyses

We will perform the following subgroup analyses:

- Per trimester of pregnancy.
- Per health zone.
- Per study site.
- Where sequencing information is available, per detected MPXV clade.

We will perform the following sensitivity analyses:

- Include women who were excluded due to having a Ct value at or above the cut-off of 40.
- Use survival curves to visualize gestational age at delivery, plotted separately per risk factor (see Table 12). We will perform the same analysis with time from hospitalization to birth outcome, and with time from symptom onset to birth outcome. Due to the small sample size, we do not plan to fit a survival analysis model.
- Compare variables related to duration of symptoms (derived variables `'Duration of hospital stay, in days'` and `'Time from symptom onset to hospital discharge, in days'`) to the characteristics of the broader study populations of MBOTE-SK and PALM007.

References

1. Van Dijck C, Hoff NA, Mbala-Kingebeni P, et al. Emergence of mpox in the post-smallpox era—a narrative review on mpox epidemiology. *Clinical Microbiology and Infection*. 2023;29(12):1487-1492. doi:10.1016/j.cmi.2023.08.008
2. Gessain A, Nakoune E, Yazdanpanah Y. Monkeypox. *New England Journal of Medicine*. 2022;387(19):1783-1793. doi:10.1056/NEJMra2208860
3. Brosius I, Vakaniaki EH, Mukari G, et al. Epidemiological and clinical features of mpox during the clade Ib outbreak in South Kivu, Democratic Republic of the Congo: a prospective cohort study. *The Lancet*. Published online January 2025. doi:10.1016/S0140-6736(25)00047-9
4. Pk M, Jw H, T RR, et al. Maternal and Fetal Outcomes Among Pregnant Women With Human Monkeypox Infection in the Democratic Republic of Congo. *J Infect Dis*. 2017;216(7). doi:10.1093/infdis/jix260
5. Vakaniaki EH, Kuispond NRS, Hirata Y, et al. Three Cases of Vertical Transmission of Clade Ib Mpox Virus. *New England Journal of Medicine*. 2025;392(23):2385-2387. doi:10.1056/NEJMc2503347
6. Nachega JB, Mohr EL, Dashraath P, et al. Mpox in Pregnancy — Risks, Vertical Transmission, Prevention, and Treatment. *New England Journal of Medicine*. Published online 2024. doi:10.1056/NEJMp2410045
7. The American College of Obstetricians and Gynecologists 409 12th Street SPB 96920, WD 20090 6920. Committee Opinion No. 700 Summary: Methods for Estimating the Due Date. Obstetrics & Gynecology . *Obstetrics & Gynecology*. 2017;129(5):967-968.
8. World Health Organization 2022. Clinical management and infection prevention and control for monkeypox: interim rapid response guidance, 10 June 2022. Published online June 10, 2022.
9. D'Antonio F, Pagani G, Buca D, Khalil A. Monkeypox infection in pregnancy: a systematic review and metaanalysis. *Am J Obstet Gynecol MFM*. 2023;5(1):100747. doi:10.1016/j.ajogmf.2022.100747
